# BMP feed-forward loop promotes terminal differentiation in gastric glands and is interrupted by *H. pylori*-driven inflammation

**DOI:** 10.1038/s41467-022-29176-w

**Published:** 2022-03-24

**Authors:** Marta Kapalczynska, Manqiang Lin, Jeroen Maertzdorf, Julian Heuberger, Stefanie Muellerke, Xiangsheng Zuo, Ramon Vidal, Imad Shureiqi, Anne-Sophie Fischer, Sascha Sauer, Hilmar Berger, Evelyn Kidess, Hans-Joachim Mollenkopf, Frank Tacke, Thomas F. Meyer, Michael Sigal

**Affiliations:** 1 Department of Hepatology and Gastroenterology, Charité University Medicine, 13353 Berlin, Germany; 2 Department of Molecular Biology, Max Planck Institute for Infection Biology, 10117 Berlin, Germany; 3 Department of Immunology, Max Planck Institute for Infection Biology, 10117 Berlin, Germany; 4 Departments of Gastrointestinal Medical Oncology, The University of Texas MD Anderson Cancer Center, Houston, TX USA; 5 Berlin Institute for Medical Systems Biology (BIMSB), Max Delbrück Center for Molecular Medicine, 10115 Berlin, Germany; 6 Laboratory of Infection Oncology, Institute of Clinical Molecular Biology (IKMB), Christian Albrechts University of Kiel, Kiel, Germany; 7 Berlin Institute of Health, 10117 Berlin, Germany

**Keywords:** Bacterial pathogenesis, Adult stem cells, Bacterial infection, Cell signalling, Gastrointestinal diseases

## Abstract

*Helicobacter pylori* causes gastric inflammation, gland hyperplasia and is linked to gastric cancer. Here, we studied the interplay between gastric epithelial stem cells and their stromal niche under homeostasis and upon *H. pylori* infection. We find that gastric epithelial stem cell differentiation is orchestrated by subsets of stromal cells that either produce BMP inhibitors in the gland base, or BMP ligands at the surface. Exposure to BMP ligands promotes a feed-forward loop by inducing *Bmp2* expression in the epithelial cells themselves, enforcing rapid lineage commitment to terminally differentiated mucous pit cells. *H. pylori* leads to a loss of stromal and epithelial *Bmp2* expression and increases expression of BMP inhibitors, promoting self-renewal of stem cells and accumulation of gland base cells, which we mechanistically link to IFN-γ signaling. Mice that lack IFN-γ signaling show no alterations of BMP gradient upon infection, while exposure to IFN-γ resembles *H. pylori*-driven mucosal responses.

## Abstract (web summary)

Helicobacter pylori causes gastric inflammation, gland hyperplasia and is linked to gastric cancer. Here the authors identify a BMP feedback loop between the stomach epithelium and surrounding stroma that controls gland homeostasis and demonstrate its interruption upon infection with H. pylori.

## Introduction

The epithelium of the gastrointestinal tract is characterized by high turnover^1–3^. In the stomach antrum, proliferative Axin2+ stem cells located in the lower isthmus of the glands give rise to the various differentiated cells that make up the rest of the gland^4^. The two major differentiated cell types are secretory cells located in the gland base and secretory pit cells.

Although almost the entire gland is renewed every one to two weeks, in the healthy state, the number and localization of the different cell types within the gland are stable over time. However, infection with the stomach bacterium *Helicobacter pylori* results in enhanced proliferation and altered differentiation^5–7^. The resulting pathological changes such as hyperplasia and atrophy represent premalignant lesions. Infection is normally accompanied by an influx of immune cells; however, how immune cells affect the behavior of stem cells and their niche is not fully understood.

We have previously shown that *H. pylori* can invade gastric glands^5^, leading to an upregulation of R-spondin 3 (Rspo3) in stromal myofibroblasts directly beneath the gland base. This enhances WNT signaling, leading to increased overall proliferation driven by Axin2+ cells in the lower isthmus as well as an expansion of the Lgr5+ compartment in the base, which contains a subset of proliferative stem cells as well as Lgr5+ secretory gland bases cells, overall resulting in hyperplasia^8^. In this way, gland-associated bacteria have a significant impact on gland homeostasis. These findings represent an important example of the role of stromal cells in epithelial homeostasis and pathology. Here, we applied a transcriptome approach to study stromal cell behavior during homeostasis and infection. We identify a spatially controlled expression pattern of BMP signaling molecules that direct epithelial differentiation along the gland axis: Stroma-derived BMP signaling near the gland surface blocks proliferation and induces rapid lineage commitment towards terminally differentiated Muc5ac+ mucous cells, while the gland base/isthmus module, consisting of secretory Lgr5+ cells and self-renewing stem cells, is preserved by BMP inhibition. Stromal cells in the lamina muscularis mucosae beneath the gland base express multiple BMP inhibitors, which block the BMP signal to allow stem cell self-renewal and proliferation. By contrast, differentiation into surface pit cells that exit the gland base is facilitated by a positive feed-forward loop that induces *Bmp2* expression in a BMP-dependent manner.

Infection with *H. pylori* is associated with increased expression of BMP inhibitors and loss of BMP ligands, resulting in an accumulation of base cells, proliferation, and hyperplasia. These effects require a functional *H. pylori* type IV secretion system (T4SS) and the inflammatory T cell cytokine IFN-γ, which inhibits *Bmp2* expression and BMP signaling in the stroma and epithelium. In addition, we find that BMP2 inhibits *Rspo3* expression in stromal cells, indicating that the loss of *Bmp2* expression upon infection is responsible for the increased expression of *Rspo3*.

Our findings reveal that gland homeostasis is regulated at multiple levels by BMP signaling and that inflammation can interfere with this regulatory network via IFN-γ, resulting in altered differentiation and gland pathology.

## Results

### Myh11+ myofibroblasts respond to *H. pylori* infection

**Fig. 1:**
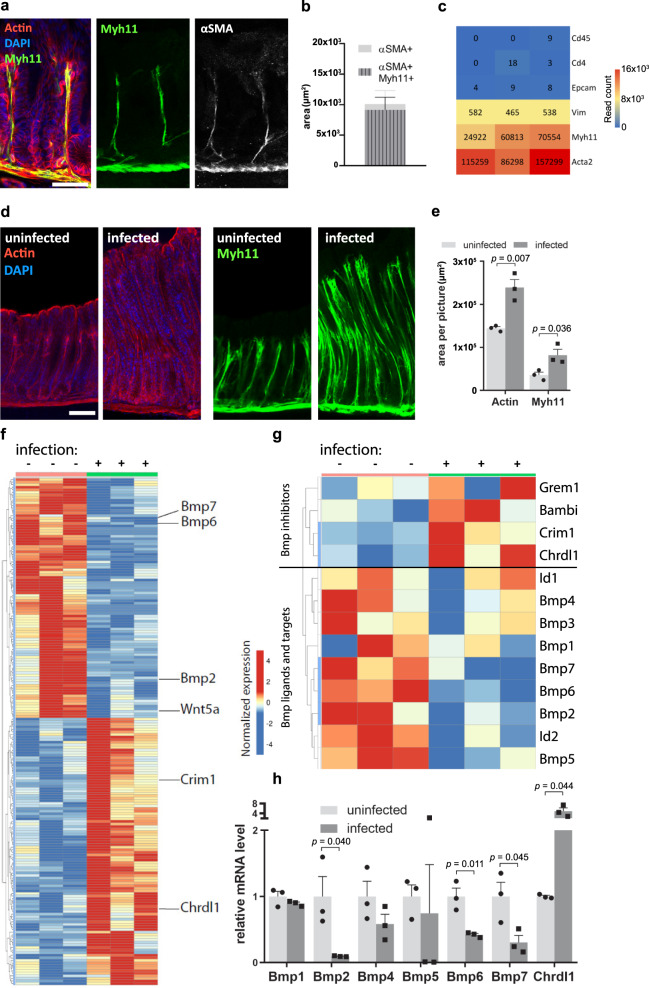
*H. pylori* infection reduces BMP signaling in Myh11+ myofibroblasts. **a** Confocal microscopy images of stomach antrum from *Myh11CreErt2/Rosa26-mTmG* mice. **b** Quantification of the signal overlap between α-SMA+ and Myh11-eGFP signal from *Myh11CreErt2/Rosa26-mTmG* mice (*n* = 3). **c** Heat map for selected genes expressed in sorted Myh11-eGFP cells from *Myh11CreErt2/Rosa26-mTmG* mice (*n* = 3). The gene expression level is presented as the read count. **d** Confocal microscopy images of gland hyperplasia (left) and Myh11+ myofibroblasts (right) in uninfected and 2-month *H. pylori*-infected *Myh11CreErt2/Rosa26-mTmG* mice. **e** Quantification of actin and Myh11 area per picture in uninfected (*n* = 3) and 2-month *H. pylori*-infected (*n* = 3) *Myh11CreErt2/Rosa26-mTmG* mice. **f** Heat map of RNAseq data with genes differentially expressed in uninfected (*n* = 3) and 2-month *H. pylori*-infected (*n* = 3) Myh11+ myofibroblasts. **g** Highlight of RNAseq data for differentially expressed genes involved in BMP signaling in uninfected (*n* = 3) and 2-month *H. pylori*-infected (*n* = 3) Myh11+ myofibroblasts. **h** qPCR validation of RNAseq data for BMP genes (uninfected: *n* = 3; infected: *n* = 3). Images are representative of at least three biological replicates. Scale bar: 100 µm. Data are presented as mean ± SEM. Statistical analyses were performed using Student’s *t*-test (two-tailed) for (**e**) and (**h**). RNAseq data are from three biological replicates per group. Source data are provided as a Source Data file.

To investigate what impact the stroma along the entire gastric gland has on gland homeostasis, we generated *Myh11CreErt2/Rosa26-mTmG* mice to induce GFP expression in the Myh11-myofibroblast lineage. Confocal microscopy identified GFP+ cells beneath and between the glands in direct proximity to epithelial cells, and staining with α-smooth muscle actin (SMA) antibody showed co-localization with the GFP signal (Fig. 1a), indicating that it is specific to mesenchymal cells. Signal quantification confirmed a strong overlap between Myh11 and α-SMA signal (Fig. 1b), and gene expression analysis from sorted gastric GFP+ cells revealed that Myh11+ cells indeed express high levels of mesenchymal markers, whereas markers of epithelial and immune cells were absent (Fig. 1c).

Next, *Myh11CreErt2/Rosa26-mTmG* mice were infected with *H. pylori* for 2 months. Infection induced gland hyperplasia, which was accompanied by an expansion of surrounding Myh11+ cells (Fig. 1d, e). Next, we asked how the infection affects gene expression in myofibroblasts. Cells isolated from the antrum and transitional zone of infected animals were sorted for Myh11+/ Epcam− cells (Supplementary Fig. 1a), which were further processed for RNA-seq. Myofibroblasts from uninfected mice expressed a variety of genes involved in the regulation of epithelial stem cells, including Wnt, Rspo, and Bmp, as well as activators and inhibitors of these pathways. Upon infection with *H. pylori*, 104 genes were significantly upregulated and 93 downregulated (Fig. 1f), indicating that myofibroblasts actively respond to infection. We noticed that several of these genes were involved in BMP signaling. Further analysis revealed that the upregulated genes included several physiological BMP inhibitors, including *Grem1*, *Bambi*, *Crim1*, and *Chrdl1*. In contrast, expression of BMP ligands, including *Bmp1–7*, as well as the BMP pathway target genes *Id1* and *Id2* was decreased (Fig. 1g). We were able to confirm a significant loss of *Bmp2*, *Bmp6*, and *Bmp7* expression, as well as a significant increase of *Chrdl1* via qPCR (Fig. 1h). Accordingly, *H. pylori* infection induces a shift towards inhibition of BMP signaling in Myh11+ myofibroblasts.

### BMP signaling is spatially organized along the gland axis

**Fig. 2:**
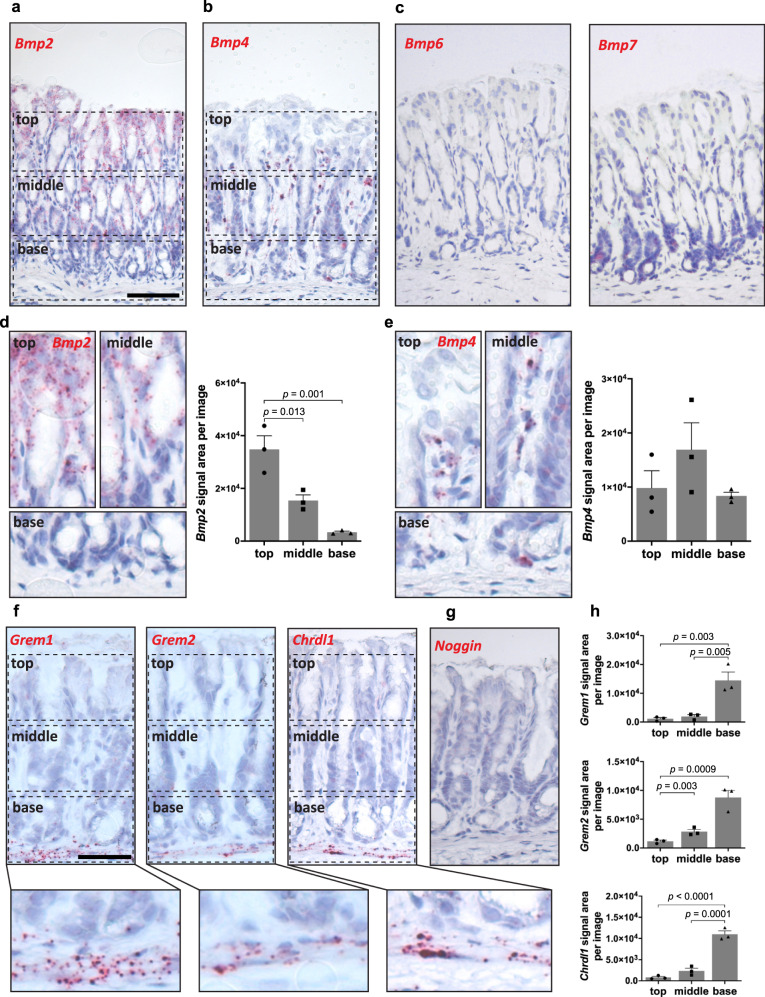
BMP signaling shows highly organized distribution in gastric glands, with strong expression in the pit cells and absence or inhibition in the gland base. **a**–**c** Single-molecule in situ hybridization (sm-ISH) for *Bmp2*, *Bmp4*, *Bmp6*, and *Bmp7* in antrum tissue (*n* = 3). **d**, **e** High magnification and quantification for *Bmp2* and *Bmp4* signal area per picture divided into top, middle, and base of the gland (*n* = 3). **f** Sm-ISH for *Grem1*, *Grem2*, and *Chrdl1* in antrum tissue with high magnification of the gland base (*n* = 3). **g** Sm-ISH for Noggin in antrum tissue. **h** Quantification for *Grem1*, *Grem2*, and *Chrdl1* signal area per picture divided for top, middle, and base (*n* = 3). Images are representative of at least three biological replicates. Scale bar: 100 µm. Data are presented as mean ± SEM. Statistical analyses were performed using one-way ANOVA, followed by Tukey’s multiple comparisons test for (**d**, **e**, **h**). Source data are provided as a Source Data file.

To visualize the spatial distribution of different BMP signaling molecules in the uninfected gastric epithelium, we performed single-molecule in situ hybridization (sm-ISH) for a number of BMP ligands. We noticed that *Bmp2*, the most highly expressed gene in both epithelial and stromal cells, was expressed predominantly at the gland surface and gradually decreased towards the base, where it was not present at all (Fig. 2a). Other BMP ligands were expressed at lower levels, e.g., *Bmp4*, which was distributed equally along the entire gland axis but was mostly restricted to the stroma and only occasionally co-localized with epithelial cells (Fig. 2b). As the expression of *Bmp6* and *Bmp7* was very low (Fig. 2c), we focused on the most abundant ligands, *Bmp2* and *Bmp4*. To quantify the distribution along the gland axis, we divided the tissue into top, middle, and base parts and quantified the signal for *Bmp2* and *Bmp4* in each. In the top of the gland, the average *Bmp2* expression was ten times higher than in the base (Fig. 2d). Quantification of the *Bmp4* signal confirmed no significant difference in expression between the top, middle, and base of the gland (Fig. 2e).

We then analyzed the spatial distribution of BMP inhibitors. ISH showed high expression of *Grem1*, *Grem2*, and *Chrdl1* in the stroma beneath the base of the glands (Fig. 2f). Noggin, which acts as a BMP inhibitor in the intestine^9^, was not detected in the stomach (Fig. 2g). Quantification of ISH showed that *Grem1*, *Grem2*, and especially *Chrdl1* expression was limited to the stromal cells beneath the gland base in the lamina muscularis mucosae (Fig. 2h). Together, these data depict a differential distribution of BMP signaling molecules along the gastric gland axis—with strong expression of *Bmp2* in the pit region and absence of *Bmp2* along with expression of BMP inhibitors in the base. To further dissect the stromal compartment in the stomach, we performed single-cell RNA sequencing (scRNA-seq) of non-epithelial cells from the gastric antrum. Unbiased clustering revealed the presence of 6 populations, which are representative for smooth muscle cells, immune cells, remaining epithelial cells, endothelial cells, and two populations of mesenchymal stromal cells that expressed the contractile gene *Acta2* (Supplementary Fig. 2a, d). To validate these results, we analyzed data from a published scRNA-seq experiment on gastric stromal cells^10^, which confirmed the presence of two mesenchymal cell populations (stromal 1 and 2) expressing the pan-fibroblast marker *vimentin, Acta2*, as well as extracellular matrix-related genes, such as *Col4a5* and *Col6a2* (Supplementary Fig. 2e, f).

Mapping of Bmp-associated genes revealed differential expression in the two stromal clusters: While *Chrdl1* and *Grem1* were almost exclusively expressed in population 2, *Bmp2* was highly expressed in population 1 (Supplementary Fig. 2b, c). Interestingly, expression of *Id1* was almost absent in the cell cluster that expressed BMP inhibitors, while the Bmp2-expressing cluster contained numerous cells expressing high levels of *Id1*, indicating that BMP signaling in the stroma is inhibited in cells surrounding the gland base. Population 1 also showed high expression of *Foxl1*, which was absent in population 2 (Supplementary Fig. 2d). Differential expression of BMP ligands and inhibitors was also consistently found in the validation data set (Supplementary Fig. 2f) and recent scRNA-seq data from the intestine^11^.

### *H. pylori* regulates BMP ligand and inhibitor expression

**Fig. 3:**
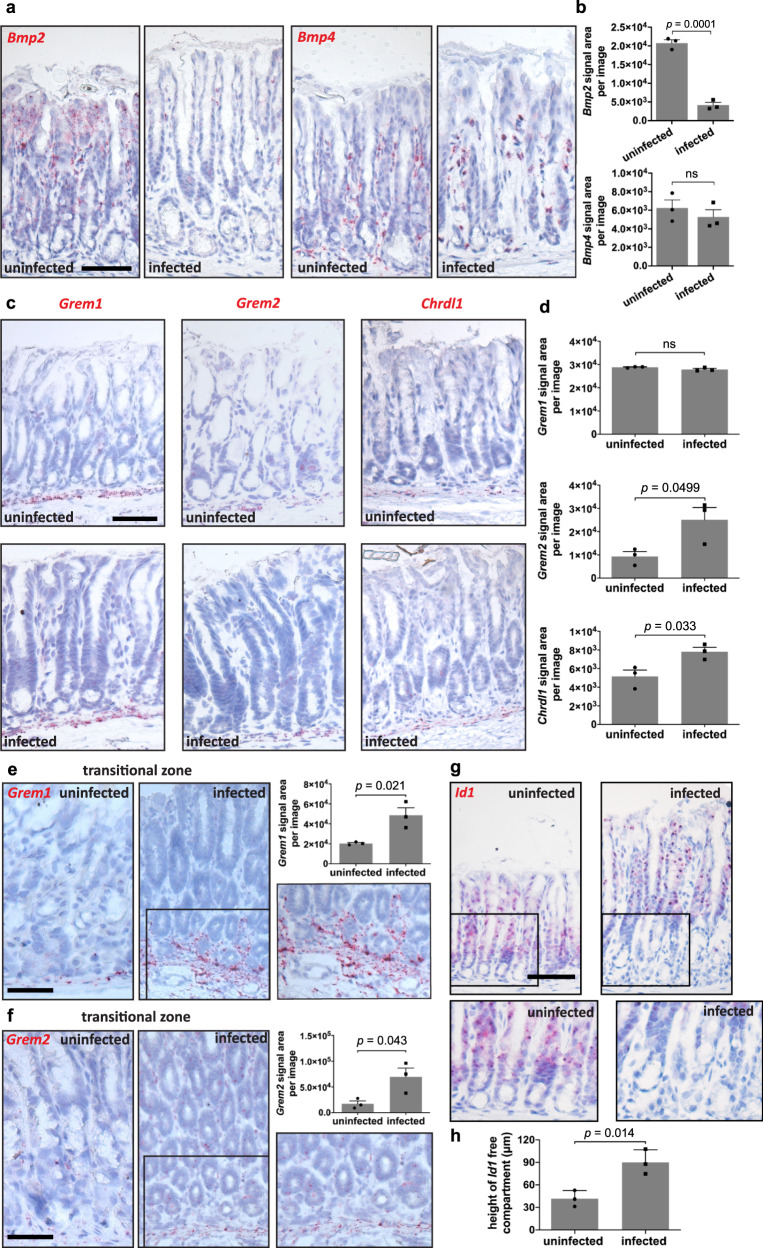
Infection with *H. pylori* reduces BMP ligand at the surface and enhances BMP inhibitors in the base of the gland. **a** Sm-ISH for *Bmp2* and *Bmp4* in antrum tissue of uninfected (*n* = 3) and 2-month *H. pylori*-infected (*n* = 3) C57BL/6 mice. **b** Quantification for *Bmp2* and *Bmp4* signal area per picture in antrum tissue of uninfected (*n* = 3) and 2-month *H. pylori*-infected (*n* = 3) C57BL/6 mice. **c** Sm-ISH for *Grem1*, *Grem2*, and *Chrdl1* in antrum tissue of uninfected (*n* = 3) and 2-month *H. pylori*-infected (*n* = 3) C57BL/6 mice. **d** Quantification for *Grem1*, *Grem2*, and *Chrdl1* signal area per picture in antrum tissue of uninfected (*n* = 3) and 2-month *H. pylori*-infected (*n* = 3) C57BL/6 mice. **e**, **f** Sm-ISH and quantification for *Grem1* and *Grem2* signal area per picture in the transitional zone between antrum and corpus of uninfected (*n* = 3) and 2-month *H. pylori*-infected (*n* = 3) C57BL/6 mice. **g**, **h** Sm-ISH for *Id1* and quantification of the *Id1*-free compartment in the gland base of uninfected (*n* = 3) and 2-month *H. pylori*-infected (*n* = 3) C57BL/6 mice. Images are representative of at least three biological replicates. Scale bar: 100 µm. Data are presented as mean ± SEM. Statistical analyses were performed using Student’s t-test (two-tailed) for (**b**, **d**–**f**, **h**). Source data are provided as a Source Data file.

To further map gene expression changes upon *H. pylori* infection in situ, we examined the distribution of BMP ligand and inhibitor transcripts in the infected mouse antrum. ISH from gastric tissue of 2-month infected mice revealed a strong decrease of *Bmp2* expression at the gland surface, in both stroma and epithelial cells, whereas *Bmp4* expression remained largely unchanged (Fig. 3a). Quantification of the signal showed an almost sevenfold lower expression of *Bmp2* in the infected samples compared to those from uninfected mice (Fig. 3b). A significant decrease of *Bmp2* expression was also confirmed in an additional set of mice infected with *H. pylori* for only 2 weeks (Supplementary Fig. 3a).

We next visualized the BMP inhibitors in the antrum and confirmed an increased expression of *Chrdl1* as well as *Grem2* in infected samples (Fig. 3c, d). *Grem1* did not show a significant difference upon infection when analyzing the whole antrum, but we noticed a striking increase of *Grem1* and *Grem2* in the proximal antrum and the transitional zone between the antrum and corpus (Fig. 3e, f). This area is particularly highly colonized by gland-associated *H. pylori*^12^ and exhibits particularly prominent gland hyperplasia and antral metaplasia. Notably, *Grem1* and *Grem2* expression was not only increased but was no longer restricted to the lamina muscularis mucosae, instead of extending upwards to the area surrounding the basal antral cells. Next, we performed ISH for *Id1* in uninfected and infected mice. We found expression of *Id1* at the surface but not at the base in uninfected mice, which is consistent with the presence of BMP inhibitors specifically at the base (Fig. 3g). Infected mice showed a significant expansion specifically of the *Id1*-free compartment at the gland base (Fig. 3g, h). Together, *H. pylori* infection downregulates *Bmp2* expression at the top of the gland and upregulates BMP inhibitors at the gland base, resulting in an expansion of *Id1*-negative epithelial cells in the bottom of gastric glands.

### BMP promotes stem cell differentiation into surface cells

**Fig. 4:**
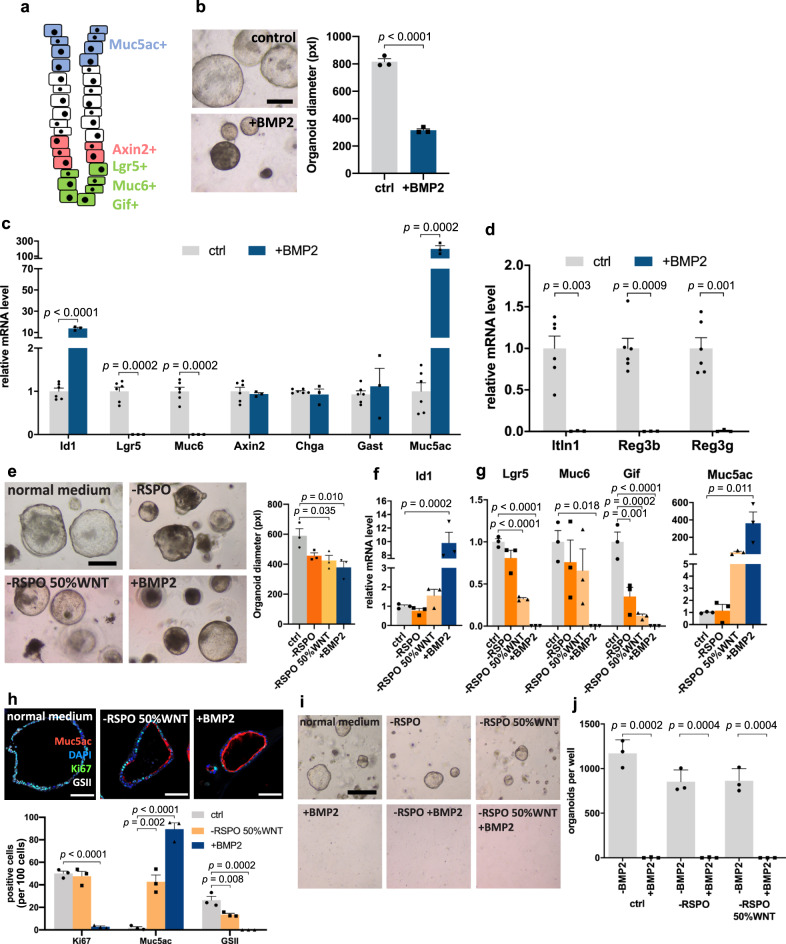
BMP promotes mucous pit cell differentiation and inhibits basal marker expression. **a** Schematic representation of gastric antrum markers for characteristic gland areas. **b** Images of organoids and quantification of the diameter of organoids from antral epithelium either untreated or treated with BMP2 (*n* = 3). **c** qPCR for the BMP target gene *Id1* and markers characteristic for specific gland regions from organoids untreated or treated with BMP2 (*n* = 3). **d** qPCR for antimicrobial genes from organoids untreated or treated with BMP2 (*n* = 3). **e** Representative pictures and diameter quantification of organoids from antral epithelium cultured in a full medium, medium without Rspo, medium without Rspo, and reduced WNT or with the addition of BMP2 protein (*n* = 3). **f** qPCR for BMP target gene *Id1* from organoids cultured in indicated conditions (*n* = 3). **g** qPCR for markers characteristic for specific gland regions from organoids cultured in indicated conditions (*n* = 3). **h** Immunofluorescence images of organoids grown in indicated conditions and quantification of relative abundance of Ki67+ cells, Muc5ac+ pit cells, and GSII+ gland base secretory cells (*n* = 3). Scale bar: 100 µm. **i**, **j** Representative organoid images and quantification of antral organoids per well treated with indicated conditions, passaged and cultured in normal medium (*n* = 3). Images are representative of at least three biological replicates. Scale bar: 250 µm. Data are presented as mean ± SEM. Statistical analyses were performed using Student’s *t*-test (two-tailed) for (**b**, **c**, **d**, **j**); using one-way ANOVA, followed by Tukey’s multiple comparisons test for (**e**, **f**, **g**, **h**). Source data are provided as a Source Data file.

The antral epithelium is characterized by a strict cellular organization: proliferative Axin2 cells in the lower isthmus either self-renew and give rise to Lgr5+ secretory cells in the base, which express markers such as Muc6 and Gif, or they lose their self-renewal capacity and differentiate into Muc5ac+ mucous pit cells in the surface region (Fig. 4a). To investigate how BMP signaling affects gastric epithelial proliferation and differentiation, we established 3D organoids from mouse antrum. The addition of recombinant BMP proteins to the medium rapidly inhibited organoid growth (Figs. 4b and S3b). However, the addition of BMP inhibitors such as CHRDL1, GREM1, and GREM2 to the culture with BMP ligands was sufficient to block the effect of BMPs, resulting in organoids that were phenotypically similar to those cultured in normal medium, as confirmed by quantification of their size (Supplementary Fig. 3b).

We then performed qPCR to examine the expression of cell type-specific markers in organoids. We first confirmed that the BMP pathway was efficiently induced by validating that expression of the BMP target gene *Id1* was strongly increased upon treatment with BMP proteins (Fig. 4c). We selected marker genes characteristic of either antrum gland base or gland surface and noticed a strong downregulation of gland base markers *Lgr5* as well as *Muc6* upon BMP treatment (Figs. 4c and S3c). In contrast, the surface pit cell marker *Muc5ac* was significantly upregulated (Figs. 4c and S3c). Expression of *Axin2* itself, as well as enteroendocrine cell markers such as gastrin (*Gast*) and chromogranin A (*Chga*), remained unchanged (Figs. 4c and S3c). BMP2 and BMP4 had a similar effect on gene expression, although *Muc5ac* expression was much higher upon BMP2 compared to BMP4 treatment (Figs. 4c and S3c). To test whether BMP antagonists are able to block the effects of BMP on differentiation, we repeated the experiment, adding CHRDL1, GREM1, or GREM2 to the medium in addition to BMP ligands, and found that all of them blocked the BMP-driven differentiation towards *Muc5ac-*expressing pit cells (Supplementary Fig. 3d).

We recently showed that secretory Lgr5+ cells produce antimicrobial factors capable of counteracting *H. pylori* infection^8^. Since expression of Bmp ligands, which inhibit *Lgr5* expression, is blocked upon infection, we wondered if the expression of antimicrobial genes is also inhibited by BMP signaling. Therefore, we examined transcription of the gland base antimicrobial factors *Itln1*, *Reg3b*, and *Reg3g* in organoids treated with exogenous BMP2 and BMP4. qPCR showed a strong decrease for all three genes upon BMP treatment (Figs. 4d and S3e). Next, we examined the influence of the three BMP inhibitors and found that all of them restored expression of *Itln1*, *Reg3b*, and *Reg3g*, compared to samples treated with BMP4 alone (Supplementary Fig. 3f). Together, these data indicate that activation of BMP signaling induces increased expression of surface mucous cell markers and that inhibition of BMP signaling promotes expression of antimicrobial genes found in the gland base upon infection.

### BMP signaling enforces rapid terminal differentiation

Our previous work showed that Rspo3-driven stabilization of WNT signaling promotes stem cell differentiation into gland base secretory cells^4^. Expression of Rspo3 is strictly restricted to the base, which correlates with the expression pattern of WNT target genes in the gland. Using organoid cultures, it has been shown that surface enterocyte markers are enriched upon removal of WNT or Rspo from the culture medium, indicating that cells that exit the gland base compartment automatically differentiate into surface cells^13^. Therefore, we compared the effects of Rspo/WNT-depletion or BMP activation on epithelial stemness, proliferation, and differentiation.

Treatment with BMP2 led to a more marked decrease in organoid size compared to a reduction of WNT ligand concentration or removal of Rspo from the medium (Fig. 4e). qPCR confirmed that the BMP target gene *Id1* was significantly upregulated after BMP2 treatment (Fig. 4f). As expected, the lack of WNT signaling, as well as the addition of BMP2, reduced the expression of gland base markers, but the effect of BMP2 was much stronger, leading to an almost complete loss of these markers (Fig. 4g). In contrast, expression of the surface cell marker *Muc5ac* was increased - here again, BMP2 had a much stronger effect (Fig. 4g). We stained organoids for Muc5ac, as well as Ki67 and GSII to visualize gland base secretory cells. While removal of WNT/Rspo induced a slight increase of Muc5ac+ cells with some Ki67+ cells still present, BMP2-treated organoids almost completely lost both the GSII+ and the Ki67+ cells, with almost 100% of cells now positive for Muc5ac (Fig. 4h). Thus, we conclude that BMP2 induces rapid terminal differentiation into surface mucous cells.

To further dissect whether this is driven by lineage commitment or death of the other cell types, we performed lineage tracing in organoids from *Lgr5CreErt2/Rosa26-tdTomato* mice (Supplementary Fig. 4a) and *Axin2CreErt2/Rosa26-tdTomato* mice (Supplementary Fig. 4b). We tracked organoids on a daily basis and confirmed that BMP2 inhibits organoid growth (Supplementary Fig. 4c). Lgr5+ or Axin2+ cell lineage tracing was induced at the same time as treatment with BMP2. We found that although organoids grew much more slowly in the presence of BMP2, there was an expansion of initially labeled clones. Quantifying the number of organoids with fluorescent signal showed that BMP2 treatment did not significantly affect the proportion that contained tdTomato+ cells, indicating that most labeled cells did not undergo cell death (Supplementary Fig. 4a, b). We conclude that induction of BMP signaling promotes rapid differentiation of stem cells into Muc5ac+ mucous cells.

Next, we passaged the organoids grown in a medium that was Rspo/WNT depleted, BMP2 supplemented, or both and transferred them back to a normal stem cell medium to assess their colony-forming efficiency. Cells kept in medium without Rspo and with reduced WNT were able to generate organoids when returned to normal medium, but cultures treated with BMP2 were not, regardless of whether they grew in normal or Rspo/WNT depleted medium before passaging (Fig. 4i, j), demonstrating that BMP2 treatment completely blocks organoid forming capacity.

**Fig. 5:**
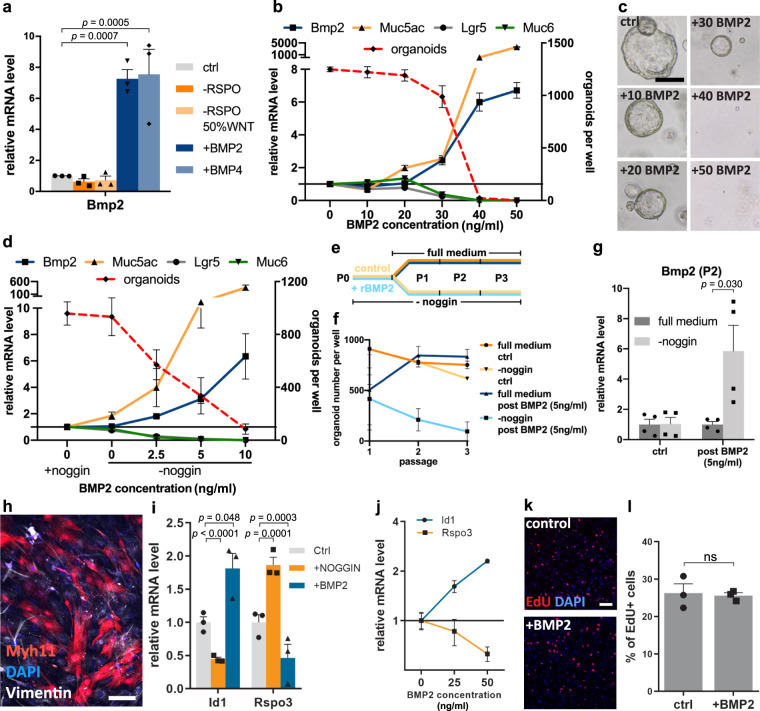
BMP signaling has a strong impact on gland turnover through the feed-forward loop and regulates R-spondin3 expression in myofibroblasts. **a** qPCR for *Bmp2* from organoids cultured in indicated conditions (*n* = 3). **b** qPCR for indicated genes and forming capacity from organoids exposed to different concentrations of rBMP2 (*n* = 3). **c** Representative images of organoids that re-grew in the full medium after exposure to the indicated rBMP2 concentrations. **d** qPCR for indicated genes and forming capacity from organoids grown without noggin, exposed to different concentrations of rBMP2 (*n* = 3). **e** Schematic representation: organoids were grown without noggin and treated with 5 ng/ml rBMP2 (blue) for 4 days at passage 0. Organoids were washed and passaged and either kept in full medium with noggin (dark blue) or without noggin (blue). **f** Organoid forming capacity at different passages from organoids treated (blue) or untreated (yellow) with rBMP. Organoids exposed to full medium with noggin (dark blue) recovered their forming capacity, while organoids without noggin progressively lost forming capacity (*n* = 2). **g** qPCR from the organoids from **e** at passage 2: *Bmp2* expression triggered by rBMP is not observed in organoids that were re-grown in full medium, while in organoids grown without noggin, endogenous *Bmp2* expression is strongly upregulated (*n* = 4). **h** Immunofluorescence image of myofibroblasts from *Myh11CreErt2/Rosa26-tdTomato* mice cultured in 2D. Scale bar: 100 µm. **i** qPCR for *Id1* and *Rspo3* from myofibroblasts cultured with noggin and BMP2 (*n* = 3). **j** qPCR for *Id1* and *Rspo3* from myofibroblasts cultured with different BMP2 concentrations (*n* = 2). **k** Immunofluorescence images of EdU proliferation assay performed with myofibroblasts untreated and treated with BMP2. Scale bar: 100 µm. **l** Quantification of the proportion of EdU positive cells (*n* = 3). Images are representative of at least three biological replicates. Scale bar: 250 µm, except where indicated. Data are presented as mean ± SEM. Statistical analyses were performed using Student’s *t*-test (two-tailed) for (**g**, **i**, **l**); using one-way ANOVA, followed by Tukey’s multiple comparisons test for (**a**). Source data are provided as a Source Data file.

Since we observed that *Bmp2* has a profound effect on epithelial differentiation and is also expressed in surface epithelial cells themselves, we investigated how its expression is controlled in the epithelium. Organoids expressed low levels of *Bmp2* when grown in the control medium as well as in a medium with reduced Rspo/WNT. In contrast, the addition of BMP2 itself, as well as its homolog BMP4, strongly upregulated *Bmp2* expression (Fig. 5a), indicative of a positive auto- and paracrine BMP feedback loop that, once activated, can be sustained and enhanced autonomously by the *Bmp2*-expressing cell compartment. By exposing organoids to different concentrations of recombinant BMP2 (rBMP2), we observed that low concentrations of rBMP2 induce a slight increase of *Muc5ac* and a slight decrease of *Lgr5* expression, resulting in reduced organoid forming capacity (Fig. 5b, c). However, once endogenous *Bmp2* is induced by rBMP2, a hyper-additive effect on all measured parameters is observed, resulting in a rapid and complete loss of *Lgr5* and *Muc6* expression as well as organoid forming capacity (Fig. 5b, c). As the normal organoid culture medium contains the BMP inhibitor noggin, we repeated this experiment in organoids grown in a noggin-free medium. Treatment with rBMP2 still had the same effects, but the concentrations required were lower (Fig. 5d). To investigate the impact of endogenous BMP2, organoids in the noggin-free medium were treated with 5 ng/ml rBMP2 for four days. Following treatment, they were washed and grown either in full medium with noggin or medium without noggin (Fig. 5e). We noticed that 5 ng/ml rBMP2 induced inhibition of organoid forming efficiency. While organoid forming efficiency could be fully recovered in cells grown with noggin at passage 2 (Fig. 5f, dark blue line), cells grown without noggin showed a progressive loss of organoid forming capacity (Fig. 5f, light blue line). This was accompanied by suppression of endogenous *Bmp2* expression in organoids grown with noggin at passage 2, while organoids previously exposed to BMP2 and grown without noggin showed an increase in *Bmp2* expression (Fig. 5g). This demonstrates that once its expression is induced, endogenous BMP2 feed-forward signaling is sufficient to enforce differentiation and loss of proliferative capacity.

We recently reported that WNT signaling in the stomach is controlled by Rspo3 expressed exclusively in myofibroblasts beneath the gland base but not in the stroma surrounding the surface of the gland^4^. Since *Bmp2* is only expressed at the gland surface, we asked whether BMP2 affects Rspo3 expression. We therefore isolated and cultured primary Myh11+ myofibroblasts from *Myh11CreErt2/Rosa26-tdTomato* mice and confirmed that they were mesenchymal cells by staining for vimentin expression (Fig. 5h). Treatment with BMP2 led to a decrease of *Rspo3* expression, while inhibition of BMP signaling by noggin increased *Rspo3* expression in myofibroblasts (Fig. 5i, j). To exclude that this is due to a selective effect on the proliferation of Rspo3+ myofibroblasts, we analyzed proliferation via the EdU proliferation assay. No significant changes in proliferation upon BMP2 treatment were observed, indicating that BMP2 signaling has a direct inhibitory effect on *Rspo3* expression in myofibroblasts (Fig. 5k, l). Thus, our data demonstrate the existence of feedback loops controlling both strength and spatial distribution of WNT and BMP signals in gastric glands.

### The deficiency of BMP signaling promotes gland hyperplasia

Smad4 is one of the central mediators through which transforming growth factor-β/BMP signaling regulates gene expression. To study if inhibition of BMP signaling in Axin2+ cells alters epithelial cell composition in the stomach, we generated *Axin2CreErt2/Smad4*^*fl/fl*^ mice. In the antrum of these mice, we did indeed find *Smad4*-depleted glands (Supplementary Fig. 4d, dashed red lines) that had almost completely lost expression of *Id1* (Supplementary Fig. 4e, dashed black lines). The KO efficiency of this model was relatively low. However, these KO glands specifically displayed an expansion of the gland base module and hyperproliferation (Supplementary Fig. 4f).

**Fig. 6:**
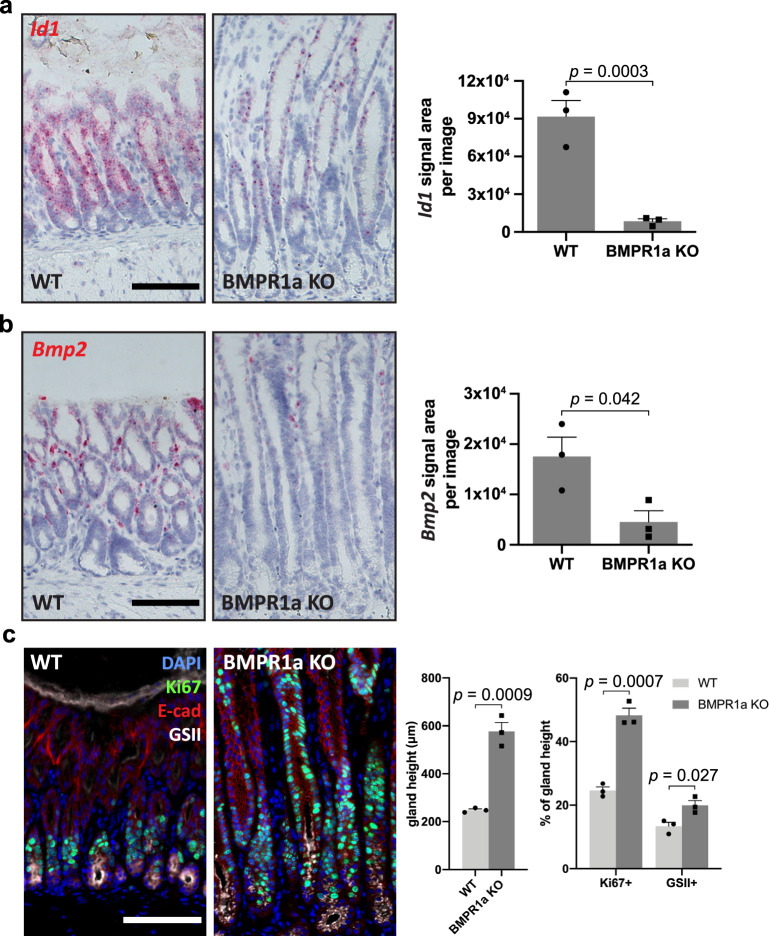
Deficiency of BMP signaling blocks the BMP feedback loop and promotes gland hyperplasia. **a** Sm-ISH for *Id1* and quantification of *Id1* signal area per image in antrum tissue from WT (*n* = 3) and *Axin2CreErt2/Bmpr1a*^*fl/fl*^ (*n* = 3) mice. **b** Sm-ISH for *Bmp2* and quantification of *Bmp2* signal area per image in antrum tissue from WT (*n* = 3) and *Axin2CreErt2/Bmpr1a*^*fl/fl*^ (*n* = 3) mice. **c** Immunofluorescence images of antrum tissue from WT (*n* = 3) and *Axin2CreErt2/Bmpr1a*^*fl/fl*^ (*n* = 3) mice. Gland height of antrum tissue and relative abundance of the Ki67+ and GSII+ compartments were quantified. Images are representative of at least three biological replicates. Scale bar: 100 µm, data are presented as mean ± SEM. Statistical analyses were performed using Student’s *t*-test (two-tailed) for (**a**–**c**). Source data are provided as a Source Data file.

Due to the low efficiency of Smad4 KO, we established another model to study the effect of BMP signaling on stem cell fate. We generated Axin2-specific BMP type I receptor Bmpr1a KO mice by breeding *Bmpr1a*^*fl/fl*^ mice with *Axin2CreErt2* mice and found that the KO in these mice was efficient. Expression of *Id1* was reduced by 90.7% (Fig. 6a), which was associated with a significant decrease of *Bmp2* expression in the glands (Fig. 6b), confirming that BMP signaling induces a positive feed-forward loop via *Bmp2*. Histological analysis revealed that KO mice had severe hyperplasia (Fig. 6c), which was characterized by an expansion of the gland base module (gland base secretory cells and proliferative cells), mimicking our observations in mice infected with *H. pylori*. We also infected KO mice with *H. pylori*. Infection caused an inflammatory response but did not augment antrum hyperplasia (Supplementary Fig. 4g), suggesting that BMP inhibition upon infection is a dominant event in the context of *H. pylori*-induced hyperplasia. Overall, these functional genetic experiments confirm that depletion of *Bmpr1a* in Axin2+ cells causes increased proliferation and accumulation of gland base secretory cells.

### *H. pylori* induces BMP2 loss and gland hyperplasia via T4SS

**Fig. 7:**
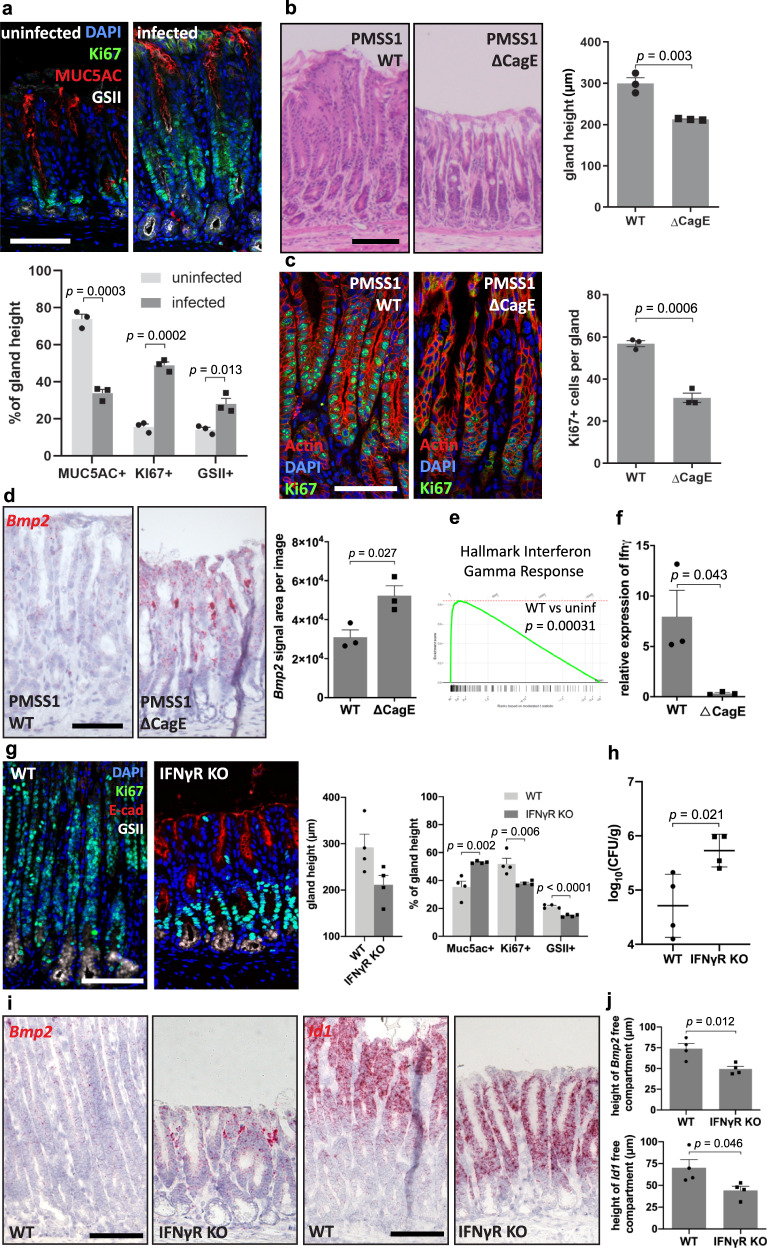
*H. pylori* induce an IFN-γ response. **a** Immunofluorescence images of antrum tissue from uninfected (*n* = 3) and 2-month PMSS1 WT *H. pylori*-infected (*n* = 3) mice. Relative abundance of the Muc5ac+, Ki67+, and GSII+ compartments were quantified. **b** Representative images of H&E staining and gland height quantification of antrum tissue from C57BL/6 mice infected for two months with PMSS1 WT (*n* = 3) or *ΔCagE H. pylori* (*n* = 3). **c** Immunofluorescence images labeled for Ki67 and quantification of Ki67+ cells per gland in antrum tissue from C57BL/6 mice infected for two months with PMSS1 WT (*n* = 3) or *ΔCagE H. pylori* (*n* = 3). **d** Sm-ISH for *Bmp2* and quantification of *Bmp2* signal area per image in antrum tissue from C57BL/6 mice infected for 2 months with PMSS1 WT (*n* = 3) or *ΔCagE H. pylori* (*n* = 3). **e** GSEA with the hallmark of IFN-γ response genes for PMSS1 WT *H. pylori*-infected (*n* = 2) compared to uninfected C57BL/6 mice (*n* = 2). **f** qPCR for *Ifnγ* in the gastric antrum from C57BL/6 mice infected for 2 months with PMSS1 WT (*n* = 3) or *ΔCagE H. pylor*i (*n* = 3). **g** Immunofluorescence images of antrum tissue from 6-week *H. pylori-infected* C57BL/6 mice (*n* = 4) and *IfnγR* KO mice (*n* = 4). Gland height of antrum tissue and relative abundance of the Muc5ac+, Ki67+, and GSII+ compartments were quantified. **h** CFUs from 6-week *H. pylori-infected* C57BL/6 mice (*n* = 4) and *IfnγR* KO mice (*n* = 4). **i**, **j** Sm-ISH images and quantification for *Bmp2* and *Id1* in antrum tissue of 6-week *H. pylori*-infected C57BL/6 mice (*n* = 4) and *IfnγR* KO mice (*n* = 4). Images are representative of at least three biological replicates. Scale bar: 100 µm, data are presented as mean ± SEM. Statistical analyses were performed using Student’s *t*-test (two-tailed) for (**a**–**d**, **f**–**h**, **j**). Source data are provided as a Source Data file.

Since we had observed that BMP signaling is inhibited upon *H. pylori* infection, we asked whether infection triggers similar changes in the glands as those observed upon BMP inhibition in organoids. Indeed, staining for cell type-specific markers revealed an expansion of GSII+ mucous gland base cells upon infection, as well as an expansion of the proliferative compartment, overall leading to gland hyperplasia, whereas Muc5ac+ cells were relatively underrepresented (Fig. 7a). These observations are consistent with our previous findings showing that infection leads to an expansion of stem cells as well as gland base secretory cells^4,8^.

The *H. pylori* T4SS is required to translocate CagA, its most prominent virulence factor, which we have previously shown to be required for the expansion of the Lgr5+ cell compartment upon infection^5^. We wondered whether the inhibition of *Bmp2* expression upon infection also depends on a functional T4SS. To address this, we infected mice with an isogenic mutant lacking the *CagE* gene, which has a dysfunctional T4SS and is not able to translocate CagA into host cells^14^. Mice infected with *ΔCagE* PMSS1 showed less gastric hyperplasia (Fig. 7b) and fewer Ki67+ cells compared to mice infected with wild-type PMSS1 (Fig. 7c). By performing ISH for *Bmp2*, we observed that the signal was still present at high levels in mice infected with *ΔCagE* PMSS1, whereas wild-type PMSS1 infected mice showed an almost complete loss of *Bmp2* expression (Fig. 7d). Thus, the downregulation of *Bmp2* in response to infection depends on a functional T4SS.

We, therefore, investigated the mechanism underlying the downregulation of *Bmp2* expression. Microarray data comparing gene expression in the antrum of uninfected versus 2-month-infected mice followed by Gene Set Enrichment Analysis (GSEA) showed the highest level of enrichment for IFN-γ response genes (Fig. 7e). Using qPCR to compare the expression of *Ifnγ* in the gastric antrum from mice infected with wild-type vs. *ΔCagE* PMSS1, we found that expression is significantly higher in wild-type-infected animals, indicating that the strong induction of *Ifn-γ* expression upon infection requires the T4SS (Fig. 7f). Thus, we conclude that translocation of CagA by *H. pylori* leads to induction of IFN-γ signaling as well as repression of *Bmp2* expression.

To investigate the role of IFN-γ for BMP-driven gastric pathology, we infected IFN-γ receptor KO (*IFNγR* KO) mice with *H. pylori*. We observed that KO mice showed less hyperplasia (Fig. 7g). Moreover, the absence of the *IFNγR* rescued infection-driven BMP alterations: we found no loss of *Bmp2* or *Id1* expression (Fig. 7i, j), but reduced *Grem2* expression in infected *IFNγR* KO mice compared to WT littermates (Supplementary Fig. 5a). While infected WT mice showed an expansion of gland base cells, this was not seen in *IFNγR* KO mice (Fig. 7g). Moreover, colony-forming unit (CFU) analysis showed higher colonization in KO mice (Fig. 7h). Overall, these data demonstrate that IFN-γ is required for the changes in BMP signaling observed after *H. pylori* infection.

*H. pylori* is known to trigger an immune response with infiltration of IFN-γ producing T cells^15^. Since we noticed that *H. pylori* already induced *Bmp2* loss after two weeks of infection (Supplementary Fig. 3a), we investigated to what extent T cells are found in the mucosa. Indeed, at two weeks, there was a strong infiltration of T cells (Supplementary Fig. 5b), indicating that the inflammatory responses and subsequent changes in epithelial cell fate determination appear at an early time point of *H. pylori* infection.

### IFN-γ inhibits BMP signaling in epithelial and stromal cells

**Fig. 8:**
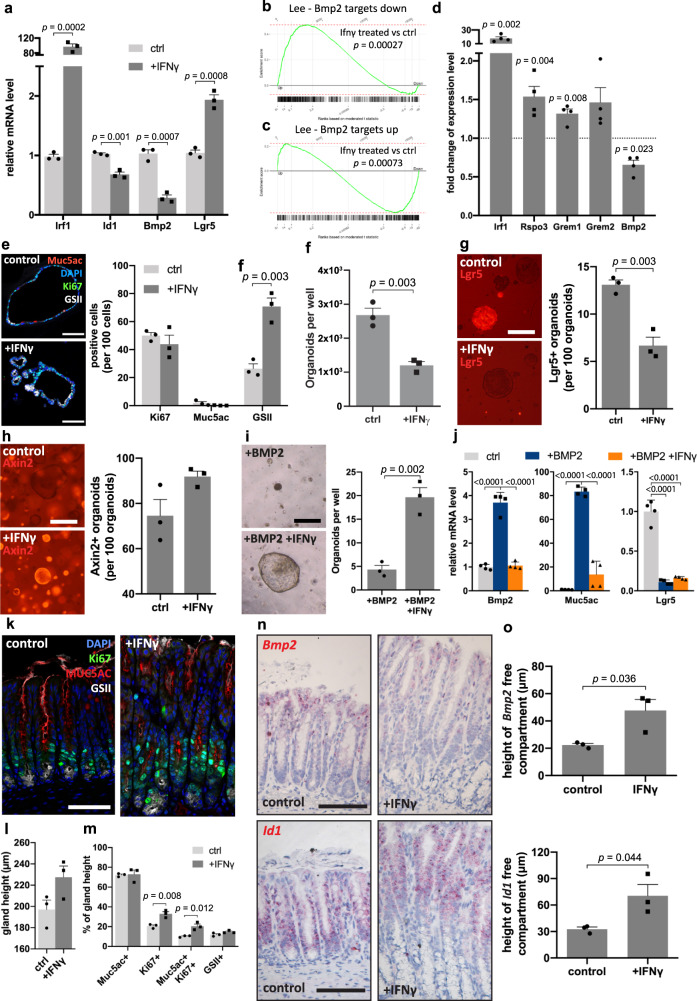
IFN-γ blocks BMP signaling. **a** qPCR for indicated genes in untreated or IFN-γ treated organoids (*n* = 3). **b**, **c** GSEA with the hallmark of targets downregulated or upregulated by BMP2 (Lee) for IFN-γ treated organoids compared to untreated ones (*n* = 2). **d** qPCR for indicated genes from primary gastric antrum stromal cells untreated or treated with IFN-γ (*n* = 4). **e** Immunofluorescence images of organoids either untreated or treated with IFN-γ; relative abundance of different cell types was quantified (*n* = 3). **f** Quantification of forming capacity of organoid either untreated or pretreated with IFN-γ (*n* = 3). **g** Images and quantification of tdTomato positive organoids from *Lgr5CreErt2/Rosa26-tdTomato* mice untreated or treated with IFN-γ, passaged and cultured in normal medium (tamoxifen was given 24 h before passaging; *n* = 3). Scale bar: 250 µm. **h** Images and quantification of tdTomato positive organoids from *Axin2CreErt2/Rosa26-tdTomato* mice untreated or treated with IFN-γ, passaged and cultured in normal medium (tamoxifen was given 24 h before passaging; *n* = 3). Scale bar: 250 µm. **i** Representative images and quantification of organoids pretreated with BMP2 or with BMP2 and IFN-γ together before passaging and then cultured in normal medium (*n* = 3). Scale bar: 250 µm. **j** qPCR for indicated genes with untreated, BMP2, or BMP2 and IFN-γ treated organoids (*n* = 4). **k** Immunofluorescence images from antrum of untreated (*n* = 3) or IFN-γ treated mice (*n* = 3). **l**, **m** Quantification of gland height and relative abundance of different cell compartments in mice upon IFN-γ treatment (ctrl: *n* = 3; +IFN-γ: *n* = 3). **n** Sm-ISH images and quantification for *Bmp2* and *Id1* in mice either untreated (*n* = 3) or treated with IFN-γ (*n* = 3). Images are representative of at least three biological replicates. Scale bar: 100 µm, except where indicated. Data are presented as mean ± SEM. Statistical analyses were performed using Student’s *t*-test (two-tailed) for (**a**, **d**–**h**, **I**, **l**, **m**, **o**); using one-way ANOVA, followed by Tukey’s multiple comparisons test for (**j**). Source data are provided as a Source Data file.

Next, we asked whether IFN-γ can directly influence the expression of BMP ligands or inhibitors in epithelial and stromal cells. We treated gastric organoids with recombinant IFN-γ and confirmed a strong upregulation of the IFN-γ target gene *Irf1* by qPCR (Figs. 8a and S5e). Interestingly, *Bmp2*, the BMP2 target gene *Id1*, as well as other target genes of BMP signaling, were downregulated upon IFN-γ treatment, while expression of *Lgr5* was increased by 85.4% (Figs. 8a, S5c, and S5e). These effects required the IFN-γ receptor, as no expression changes were observed in organoids from IFN-γ receptor KO mice (Supplementary Fig. 5d). To investigate this further, we performed microarray analysis from organoids treated with IFN-γ followed by GSEA. IFN-γ led to a significant enrichment of genes that were previously identified as being downregulated by BMP2 (Fig. 8b), whereas genes upregulated by BMP2 showed significant negative enrichment (Fig. 8c). β-catenin target genes were not significantly regulated, indicating that the IFN-γ-driven upregulation of *Lgr5* expression was driven by inhibition of BMP2 signaling and not by interfering with the WNT pathway (Supplementary Fig. 5f). As we had observed inhibition of *Bmp2* expression in stromal cells upon infection, we now asked whether this, too, was induced by IFN-γ. Indeed, we found a significant decrease of *Bmp2* expression in isolated stromal cells that were treated with IFN-γ (Fig. 8d), indicating that both epithelial and stromal BMP signaling is inhibited by IFN-γ. Since Bmp2 signaling inhibits stromal Rspo3, we asked how its expression is affected by IFN-γ and found that *Rspo3* was indeed upregulated in stromal cells exposed to IFN-γ (Fig. 8d). Moreover, expression of *Grem1* and *Grem2* was increased, consistent with the data from stromal cells of mice infected with *H. pylori* (see Fig. 3c–f).

To further investigate the direct effects of IFN-γ on gastric epithelial cells, we stained organoids treated with IFN-γ and observed enrichment of GSII+ cells, while Muc5ac+ cells were lost (Fig. 8e). Although in our organoids, IFN-γ induced higher levels of *Lgr5* expression and blocked BMP2 signaling, organoid growth was not increased upon treatment. Instead, IFN-γ treated organoids appeared slightly smaller, and fewer organoids formed after passaging (Fig. 8f).

As Lgr5 is expressed in gland base secretory cells as well as in a subset of proliferative stem cells, we asked how IFN-γ affects these subpopulations. Lineage tracing was induced with tamoxifen in IFN-γ-treated and non-treated organoids from *Lgr5CreErt2/Rosa26-tdTomato* mice. After 24 h, organoids were dissociated into single cells and passaged to determine the proportion of tdTomato positive organoids formed, i.e., cells that derive from Lgr5+ cells or their early progeny (Fig. 8g). Upon treatment with IFN-γ, the proportion of organoids deriving from tdTomato+ cells was decreased, indicating a selective advantage for organoid-forming capacity of cells that were Lgr5-negative cells compared to Lgr5-positive cells or their immediate progeny (Fig. 8g). Thus, we conclude that IFN-γ prevents the proliferation of Lgr5+ cells, which are likely to be programmed into gland base secretory cells. In contrast, when we performed the same experiment using *Axin2CreErt2/Rosa26-tdTomato* mice, in which both Lg5+ gland base cells and proliferative isthmus stem cells are labeled, we found that the proportion of Axin2+ cell-derived organoids was slightly higher upon IFN-γ-treatment (Fig. 8h), suggesting that stem cell capacity, in general, is maintained in Axin2-positive cells. We next treated organoids with BMP2 and IFN-γ to address whether IFN-γ affects stem cell differentiation under these conditions. We quantified the organoid forming efficiency when organoids were passaged, and a defined number of cells was re-plated in the regular organoid medium. BMP2 alone led to an almost complete loss of organoid forming capacity, which could be partially rescued by IFN-γ, although the absolute numbers were still much lower compared to untreated controls (Fig. 8i). Moreover, BMP2-driven overexpression of *Muc5ac* and *Bmp2*, but not loss of *Lgr5*, were inhibited by IFN-γ (Fig. 8j). Together, these data demonstrate that IFN-γ inhibits BMP2 signaling, shifting cell fate towards phenotypes present in the gland base, including mucous gland base cells and proliferative stem cells.

To address whether these effects of IFN-γ are also observed in vivo, we obtained gastric tissue from mice treated with IFN-γ for 14 days. Consistent with previous results, IFN-γ induced gland hyperplasia (Fig. 8k, l). Immunofluorescence revealed an expansion of Ki67+ cells in the base/isthmus, as well as increased numbers of Ki67+/Muc5ac+ cells (Fig. 8k, m). In situ hybridization for *Bmp2* and *Id1* revealed an expansion of cells at the base that express neither *Id1* nor *Bmp2* (Fig. 8n, o). Moreover, mice that were treated with IFN-γ expressed significantly higher levels of *Grem2* in the stroma (Supplementary Fig. 5g), similar to the mice infected with *H. pylori* (Fig. 3c, d). Together, we conclude that IFN-γ inhibits BMP signaling in the stomach in both epithelial and stromal cells. This expands the gland base module by promoting stem cell self-renewal and accumulation of gland base secretory cells.

## Discussion

Here, we show that inflammation can play a causative role in the development of hyperplasia. Inflammation-associated IFN-γ has a direct effect on BMP signaling within the gastric epithelium, which in turn controls the balance between stem cell self-renewal and differentiation. We find that the stromal cell niche that surrounds the glands exhibits positional differences along the gland axis to control the differentiation of epithelial cells on their way to the surface. Stem cell self-renewal is maintained by the expression of BMP signaling inhibitors from the stroma surrounding the base, while cells that exit the stem cell compartment are exposed to BMP signaling molecules produced by stromal cells higher up along the gland axis, which in turn induce rapid differentiation of stem cells into Muc5ac+ surface pit cells. This differentiation process is further reinforced by a positive BMP2 feed-forward loop, which independently maintains BMP2 signaling within the epithelial cells themselves and which rapidly restricts fate determination and induces loss of stemness.

That this master regulator of epithelial homeostasis can be modified by IFN-γ signaling in the context of inflammation provides a direct mechanism for the hyperproliferation and altered differentiation seen in the context of *H. pylori* infection and potentially also multiple other types of gastrointestinal injury and infection^16–19^.

BMP signaling is known to be an important mechanism for controlling differentiation in the gastrointestinal tract^20–22^. Recent studies in the intestine have revealed stromal subpopulations regulating epithelial signaling, with “trophocytes” producing BMP inhibitors, while another population was shown to be the main source of BMP ligands^11^. Our single-cell analysis also identifies mesenchymal populations with opposing expressions of BMP signaling molecules in the stomach.

Most available information on the role of BMP signaling derives from artificial models that use overexpression or inhibition in genetically modified mice: In the stomach, inhibition of BMP signaling by overexpression of noggin in parietal cells leads to hyperplasia as well as loss of parietal cell differentiation^23^. Correspondingly, BMP inhibition leads to increased expression of the stem cell marker *Lgr5* in the stomach^24^. In addition, inhibition of BMP signaling in stromal cells was shown to increase gastric epithelial proliferation via mechanisms that remained unclear^25,26^. In particular, these studies were not designed to determine the spatial restriction and endogenous regulation of this pathway. We show here a strict spatial organization of BMP signaling molecules in gastric tissue and provide mechanistic data on how this organization is maintained. Of particular relevance, we find that BMP2 induces its own expression, which maintains and augments BMP signaling and differentiation of epithelial cells towards surface pit cells. Based on our data, *Bmp2* is expressed in epithelial as well as stromal cells, and inhibition of BMP signaling in either cell type likely results in loss of the positive feedback loop, leading to the hyperplasia observed in both of these previous models.

The effects of BMP signaling in the gastrointestinal tract were initially postulated to be driven by inhibition of WNT^9^. However, a recent report showed that the BMP-driven effects on Lgr5 cells are WNT independent^24^. While multiple genes, including *Lgr5*, are regulated by both pathways, BMP-driven Smad proteins directly interfere with the promoter regions of these genes^24^. We have previously shown that Rspo3 augments WNT signaling in the stomach^4^. Depletion of *Rspo3* in stromal myofibroblasts leads to a loss of WNT signature genes, while increased expression results in an expansion of the stem cell compartment^4^. *Rspo3* expression is strictly limited to the myofibroblasts beneath the gland base and, as we demonstrate here, is inhibited by BMP2, which may explain its spatial restriction to this region. While BMP and WNT signaling may independently affect the expression of stem cell-associated genes such as *Lgr5*, our data indicate that on the tissue level, BMP inhibits WNT signaling by interfering with stromal *Rspo3* expression.

*H. pylori* infection represents the most important risk factor for the development of gastric cancer^27^. The bacteria colonize the protective mucus layer that covers the epithelium, and while the majority is free swimming^28^, a subpopulation is able to penetrate deep into the glands and attach to the intercellular junctions of various specialized cells, including stem and progenitor cells^5^. This subpopulation has been shown to be responsible for the development of epithelial pathology^5^. We have previously demonstrated that gland base cells expand upon infection, driven by an expansion of stromal *Rspo3*, but it remained unclear how this response is regulated^4^. Here we show that infection not only alters Rspo signaling in the gland but also BMP signaling. In particular, *Bmp2* expression is inhibited in hyperplastic glands, which are characterized by an expansion of the stem and progenitor cell compartment and a reduced number of differentiated surface cells. Our data indicate that loss of *Bmp2* upon infection drives the increased expression of *Rspo3*, which expands from the stroma around the base to that higher up along the gland axis. It also shows how the changes in these two signals act in concert to induce a regenerative, plastic epithelial state.

Direct infection of epithelial organoids in vitro by *H. pylori* has been shown to have no effect on *Bmp2* expression^29,30^. Instead, our data demonstrate that *Bmp2* expression is regulated by IFN-γ, a key cytokine produced by inflammatory T cells. IFN-γ is induced by the highly virulent strains of *H. pylori*^31^ and plays an important role in the development of *H. pylori*-driven epithelial pathology^14^. Accordingly, mice that lack IFN-γ do not develop gland hyperplasia or metaplasia in response to infection^32^ or in a model of autoimmune atrophic gastritis^33^. The latter shows that IFN-γ also drives epithelial pathology in other inflammatory conditions; however, the underlying mechanisms were not fully understood until now. We demonstrate here that IFN-γ signaling is induced in a T4SS-dependent manner and blocks *Bmp2* expression. This shifts gland homeostasis away from differentiation into surface pit cells and towards increased self-renewal, leading to an accumulation of gland base and isthmus cells, which on the tissue level presents as hyperplasia. Accordingly, inhibition of BMP signaling in the stomach^26,34^ resembles the IFN-γ-dependent pathology induced by *H. pylori*^32^. It also explains why strains that lack the cagPAI pathogenicity island normally evoke less severe gastric pathology. CagPAI encodes the bacterium’s CagA virulence factor as well as the T4SS required to translocate it into host cells. This induces activation of NF-κB, followed by recruitment of immune cells to the site of infection^14^. Our data suggest that this large influx of IFN-γ-producing immune cells is a crucial factor that drives hyperplasia during infection and that all these effects are rescued by knocking out the IFN-γ receptor. In addition to the enhanced proliferation, we noticed that BMP inhibition also leads to increased expression of different antimicrobial genes in the epithelium. Thus, the increased expression of BMP inhibitors may also contribute to the antimicrobial responses to infection, which we have shown to be induced in a T4SS-dependent manner^8^.

IFN-γ has previously been studied in the context of gastric pathology^35^. Experiments with gastric organoids have demonstrated parietal cell death in the corpus and it has been suggested that this leads to a regenerative response and development of metaplasia^33^. Moreover, previous in vivo findings have shown that IFN-γ is required for increased gland proliferation and hyperplasia^31^. Our data show that IFN-γ directly affects gastric stem cell behavior (e. g. promoting differentiation of Lgr5+ cells into gland base secretory cells, while simultaneously increasing self-renewal and proliferative capacity of Axin2+ isthmus cells). In addition, we find that IFN-γ not only has direct effects on epithelial fate determination but also interferes with stromal stem cell niche signaling by downregulating the expression of BMP signaling molecules, which in turn leads to upregulation of Rspo3 in the stroma, overall expanding the pro-regenerative stem cell niche.

These observations may explain not only the expansion of gland base cells in the context of *H. pylori* infection but also the reprogramming of the epithelium into regenerative states in other injury systems in the gastrointestinal tract^16–19,36,37^. Increased proliferation, hyperplasia, and altered epithelial differentiation are observed in multiple inflammatory disorders in the gastrointestinal tract. In the colon, loss of goblet cells and crypt elongation is a feature of ulcerative colitis^38^. Epithelial differentiation is also impaired in celiac disease, leading to crypt hyperplasia and villous atrophy^39^. We have recently shown that in the context of experimental chemical colitis, differentiated cells are recruited to the stem cell pool to achieve wound healing, while differentiation is impaired^36^. Here, we demonstrate a communication route between the immune system and the epithelium with its mesenchymal niche that enables an expansion of regenerative cells as a response to injury, but may also account for the altered differentiation and increased risk for carcinogenesis that is observed in the context of chronic inflammation.

It should be noted that our findings have some limitations. We identify stromal responses to infection. It will be important to explore in the future whether these changes are due to relative shifts in cellular abundance between the different stroma cell subtypes, infiltration of new cells or whether inflammatory cytokines can directly reprogram cells from BMP-expressing to BMP inhibitor-expressing cells. Moreover, our study focuses on a murine model and does not reveal whether the molecular changes observed here are also responsible for the progression of gastric epithelial pathology towards gastric cancer in humans. Thus, further investigations of the regulation and complex interplay of epithelial stem cells and their niches in the context of human *H. pylori*-driven pathology such as foveolar hyperplasia, metaplasia as well as a progression towards cancer will be important.

## Methods

### Mouse experiments

All animal experiments were performed with guidance and approval of institutional, local, and national legal authorities (LaGeSo Berlin) at the Max Planck Institute for Infection Biology and Charité Universitätsmedizin Berlin. C57BL/6 mice were obtained from Charles River Laboratory; *Myh11CreErt2* mice^40^ were a gift from Stefan Offermanns; *IfnγR* KO mice^41^, *Rosa26-mTmG*^42^ and *Rosa26-tdTomato*^43^ reporter mice were described previously. *Lgr5CreErt2/Rosa26-tdTomato* mice were generated by breeding of *Lgr5–eGFP–IRES-CreErt2 (Lgr5eGFP)*^3^ to *Rosa26-tdTomato* mice. *Axin2CreERT2/Rosa26-tdTomato* mice were generated by breeding of *Axin2CreErt2*^44^ to *Rosa26-tdTomato* mice. *Smad4*^*fl/fl*^ mice were obtained from Jackson laboratories. *Bmpr1a*^*fl/fl*^ mice were from the laboratory of Yuji Mishina^45^. All animals were maintained in autoclaved micro-isolator cages and provided with sterile drinking water and chow ad libitum. The mice were bred at the animal care facility on a 12-h light/12-h dark cycle in a controlled temperature (22.5 ± 2.5 °C) and humidity (50 ± 5%) environment. Male, 4–8 weeks old mice were used for this study. Mice were randomly assigned to experimental groups.

*Myh11CreErt2/Rosa26-mTmG* heterozygotes were intraperitoneally injected with one dose of tamoxifen (Sigma; 4 mg per 25 g body weight, diluted in 200 µl corn oil) for Cre-driven fluorophore expression in the Myh11+ cell lineage and sacrificed after 2 days. To assess the effects of IFN-γ in vivo, mice were intraperitoneally injected with 15 units/day of mouse recombinant IFN-γ (Thermo Fischer Scientific) for 21 consecutive days, as described previously^46^.

For tissue collection, the forestomach was removed and the glandular stomach was opened along the lesser curvature and flattened on paper. Stomach contents were removed and tissue was divided into three parts along the greater curvature. To minimize sampling error, in each experiment the right part of the stomach was used for confocal analysis, the central part for single-molecule RNA ISH or histopathology, and the left part for CFU analysis.

### *H. pylori* infection and CFU analysis

A 6- to 10-week old mice were intragastrically infected with 10^8^ bacteria of *H. pylori* strain PMSS1 or an isogenic mutant lacking the *CagE* gene, and sacrificed after 2 months, as previously described^5^.

The weight of stomach sections was determined and tissue was mechanically homogenized in the brain–heart infusion medium. Homogenates were plated on blood agar in serial dilutions. After several days, bacterial colonies were counted and expressed as CFU per gram of stomach. The investigators were blinded for CFU analysis.

### Histology and imaging

For confocal microscopy, stomach sections were fixed with 4% paraformaldehyde (PFA) for 1 h, washed 3× with PBS, and embedded in 4% agarose (Biozym) as described previously^5^. A 300 µm thick longitudinal section were prepared with a vibrating blade microtome (Leica). For staining of myofibroblast 2D cultures, cells were seeded on bovine collagen type 1 (Gibco) coated coverslips 24 h before fixation. Next, myofibroblasts were fixed with 4% PFA for 20 min and washed with PBS. For staining of 3D organoid cultures organoids were fixed with 2% PFA for 1 h and washed with PBS. The sections and cultured cells were permeabilized in PBS with 3% BSA, 1% saponin, and 1% Triton X-100 prior to staining. The samples were incubated overnight with primary antibodies, followed by 2 h incubation with secondary antibodies and counterstained with DAPI for visualization of nuclei and with phalloidin for visualization of cell boundaries. The following antibodies were used: rabbit anti-αSMA (Abcam, ab5694, 1:100); rabbit anti-vimentin (D21H3) (Cell Signaling Technology, 5741S, 1:100); rabbit anti-Ki67 (D3B5) (Cell Signaling Technology, 9129S, 1:100); mouse anti-Muc5ac (45M1) (Invitrogen, 12178, 1:100); mouse anti-SMAD4 (B-8) (Santa Cruz, sc-7966, 1:100); mouse anti-E-cadherin (clone 36) (BD, 610181, 1:200); rabbit anti-CD3 (SP7) (Abcam, ab5694, 1:200); DAPI (Roche, 10236276001, 1:300); Alexa Fluor 647-conjugated lectin GSII (Thermo Scientific, L32451, 1:100); Alexa Fluor 564 and Alexa Fluor 647 fluorophore-conjugated phalloidin (Life Technologies, A12381 and A22287, 1:300). Confocal laser scanning was done on Leica SP8 confocal microscope. Images were acquired with Leica Application Suite X (LAS X) and CellSensEntry (Olympus); images were analyzed with ImageJ 1.52.

### Tissue processing

Longitudinal sections were fixed in 4% PFA overnight and sent for paraffin embedding, sectioning, and staining with hematoxylin and eosin to the Charité Core Unit Immunopathology for Experimental Models.

### Myh11+ cell isolation, sorting, and RNAseq

For RNAseq analysis, *Myh11CreErt2/Rosa26-mTmG* mice were infected with *H. pylori* for 2 months and uninfected littermates were used as controls (*n* = 3 mice per group). To confirm successful colonization, a section of stomach tissue was used for CFU analysis as described above.

Stromal cell isolation was performed as described previously^47^. Briefly, the stomach was opened along the lesser curvature and the whole antrum and transitional zone were removed and washed in PBS. Epithelial cells were removed by incubating tissue in calcium and magnesium-free DMEM (Gibco) containing 10 mM EDTA for 20 min at 37 °C with gentle agitation. Next, stomach pieces were washed and incubated in DMEM (Gibco) containing Liberase TL (1 unit/ml; Roche) and DNase I (1 unit/ml; Invitrogen) at 37 °C. After 20 min, the digested fraction was collected and put on ice. This cycle was repeated two more times for a total digestion time of 60 min. The remaining stomach fragments were collected, filtered through a 100-μm mesh, and mixed with the collected supernatants.

To obtain Myh11+ cells, stromal cells were analyzed for eGFP expression by flow cytometry (instrument: BD FACSAria II Flow Cytometer; software: BD FACSDiva v 8.0.1). Cells were gated for EPCAM (APC-conjugated rat anti-EPCAM (caa7-9G8), Miltenyi Biotec, 130-123-810, 1:50) and PI (Sigma, P4864, 1:400) double negative staining. Single viable cells were gated by forward scatter and pulse-width parameters. Cells were sorted into RNeasy Plus lysis buffer from RNeasy Plus Micro Kit (Qiagen, 74034) and an RNA extraction procedure was performed according to the manufacturer’s protocol. Quality control and quantification of total RNA were carried out using an Agilent 2100 Bioanalyzer (Agilent Technologies).

For gene expression analyses of sorted cells, RNAseq libraries were generated as follows: messenger (m)RNA was reverse transcribed and pre-amplified using the SMART-Seq v4 ultra-low input RNA kit (Takara Clontech, 6348), followed by library generation using a Nextera XT DNA library prep kit (Illumina, FC-131). Sequencing of the libraries was done on an Illumina HiSeq 1500 machine.

### Microarray analysis

For microarray analysis, RNA from organoids treated with IFN-γ was isolated using the GeneJET RNA Purification Kit (Thermo Scientific, K0732) according to the manufacturer’s protocol. Microarray experiments were performed as independent dual-color dye hybridizations using two biological replicates as described previously^4^. Quality control and quantification of total RNA were assessed using an Agilent 2100 Bioanalyzer (Agilent Technologies) and a NanoDrop 1000 UV–Vis spectrophotometer (Kisker). RNA labeling was performed with the dual-color Quick-Amp Labeling Kit (Agilent Technologies). In brief, mRNA was reverse transcribed and amplified using an oligo-dT-T7 promoter primer, and the resulting cRNA was labeled with cyanine 3-CTP or cyanine 5-CTP. After precipitation, purification, and quantification, 1.25 μg of each labeled cRNA was fragmented and hybridized to whole-genome mouse 4 × 44k multipack microarrays (Agilent-014868, whole mouse genome 4 × 44K microarray kit) according to the supplier’s protocol (Agilent Technologies). Scanning of microarrays was performed with 5 μm resolution using a G2565CA high-resolution laser microarray scanner (Agilent Technologies) with an extended dynamic range (XDR). Microarray image data were analyzed with the Image Analysis/Feature Extraction software G2567AA v. A.11.5.1.1 (Agilent Technologies) using default settings and the GE2_1105_Oct12 extraction protocol. The extracted MAGE-ML files were analyzed further with the Rosetta Resolver Biosoftware, Build 7.2.2 SP1.31 (Rosetta Biosoftware). Ratio profiles comprising single hybridizations were combined in an error-weighted fashion to create ratio experiments. A 1.5-fold change expression cut-off for ratio experiments was applied together with anti-correlation of dye-swapped ratio profiles, rendering the microarray analysis highly significant (*P* < 0.01), robust, and reproducible^48^.

### Single-cell RNAseq from stromal cells

Tissue was isolated from five C57BL/6 mice and dissociated as described for stromal bulk sequencing, then labeled with an antibody against EPCAM. EPCAM-negative cells were submitted for scRNAseq and 592 cells were analyzed. R package Seurat v3 was used for the whole pipeline, using a cut-off of at least 200 genes in a single cell and a maximum of 20% mitochondrial reads. Normalization was carried out using the scTransform function including a mitochondrial percentage regression. A UMAP dimension reduction was applied using the first 15 PCA dimensions. Seurat FindClusters function was performed with a resolution of 0.2.

For validation of findings, the second set of gastric stromal scRNAseq data from a previously published experiment were re-analyzed^10^. Gene expression Ontology (GEO) accession number for the source data is GSE116514. Datasets were filtered (>50 transcripts, <5% mitochondrial reads per cell) and log-normalized.

### Single-molecule RNA in situ hybridization

For single-molecule in situ hybridization, 5 μm thick tissue sections were used. RNA in situ detection was performed with the RNAscope Red Detection Kit according to the manufacturer’s protocol (Advanced Cell Diagnostics, 322360). Positive and negative control probes were used for each experiment according to the manufacturer’s instructions. For individual genes, the following probe target regions were used: for *Bmp2* 854–2060, for *Bmp4* 586–1673, for *Bmp6* 1001–2389, for *Bmp7* 2637–3585, for *Chrdl1* 406–1469, for *Grem1* 398–1359, for *Grem2* 231– 826, for *Nog* 78–1774, for *Id1* 2–705, for *Ppib* (positive control) 98–856.

Quantification analysis was performed with at least three biological replicates per condition. ImageJ software with image deconvolution for Fast Red signal selection was used to automatically calculate the number of molecules per image. ISH for the positive control (*Ppib*) was performed and the signal was quantified, showing a similar quantity between uninfected and infected mice (Supplementary Fig. 1b).

To highlight differences between the base and the top of gastric glands, a specific image area was selected and the signal within the area was calculated automatically.

### Organoid culture

To generate organoids, antrum tissue was incubated in PBS with 0.5 mM DTT and 3 mM EDTA for 90 min. Dissociated glands were washed with PBS, mixed with 50 µl Matrigel (BD), and plated in 24-well plates. After polymerization of Matrigel, murine gastric culture medium (Advanced Dulbecco’s Modified Eagle Medium/F12 supplemented with B27, N2 (Invitrogen), N-acetyl cysteine (Sigma) penicillin/streptomycin containing 50 ng/ml epidermal growth factor, 100 ng/ml noggin, 100 ng/ml fibroblast growth factor 10, 10 nM gastrin, Rspo1-conditioned medium, and Wnt3A-conditioned medium) was overlaid. The medium supplemented with growth factors was replaced every 2–3 days. Noggin was removed for the study of BMP-inhibitors, as well as in indicated cases for specific experiments to study the role of endogenous Bmp expression.

For indicated treatments, organoids were cultured in the murine gastric medium for 2 days followed by 4 days culture with mouse recombinant proteins: 50 ng ml^−1^ BMP2 (R&D, 355-BM-010), 30 ng ml^−1^ BMP4 (R&D, 5020-BP-010), 500 ng ml^−1^ GREM1 (R&D, 956-GR-050), 500 ng ml^−1^ GREM2 (R&D, 2069-PR-050), 300 ng ml^−1^ CHRDL1 (R&D, 1808-NR-050), or 100 ng ml^−1^ IFN-γ (R&D, 485-MI-100). For treatment with BMP2 and IFN-γ together, the concentration of recombinant BMP2 was reduced to 20 ng ml^−1^ in all conditions. The medium supplemented with growth factors was replaced every 2-3 days and new doses of recombinant proteins were added. For RSPO and WNT withdrawal, factors were removed or reduced in the medium at the beginning of the organoid culture. To visualize *Lgr5* or Axin2 lineages in the organoids, 4OH-tamoxifen was added to cultures from *Lgr5CreErt2/Rosa26-tdTomato or Axin2CreErt2/Rosa26-tdTomato* mice at the indicated time points. Cultures were fixed with 4% PFA and analyzed by confocal microscopy as described above.

### Stromal cell culture

Gastric stromal cells were isolated based on a previously established protocol^47^ with further modification. Details in procedures of stromal cell isolation were described above (see “Myh11+ cell isolation, sorting, and RNAseq”). The isolated stromal cells were seeded on 12- or 6-well plates, and cultured for 2 passages in mesenchymal stem cell medium (Advanced Dulbecco’s Modified Eagle Medium/F12 supplemented with 10% FCS, 10% penicillin/streptomycin, and 10 μM Y-27632) before specific experiments. The cell identity was determined by stromal cell-like morphology and high expression of vimentin.

The specific treatments were started 1–2 days after passage. For BMP2 treatments, stromal cells at passage 2 were cultured with 30 ng ml^−1^ mouse recombinant BMP2 protein (R&D, 355-BM-010) for 6 days, unless indicated otherwise. For noggin treatments, stromal cells at passage 2 were cultured with 100 ng ml^−1^ noggin for 6 days. For IFN-γ treatments, stromal cells at passage 2 were cultured with 500 ng ml^−1^ IFN-γ for 6 days. The medium supplemented with growth factors was replaced every 2–3 days and new doses of recombinant proteins were added.

For proliferation assay, cells were seeded on bovine collagen type 1 (Gibco) coated coverslips 24 h before fixation. The EdU assay was performed with the Click-iT EdU Alexa Fluor 594 Imaging Kit according to the manufacturer’s protocol (Life Technologies, C10339). Quantification analysis was performed with at least three biological replicates per condition. Volocity 6.3 software (Quorum Technologies) was used to automatically calculate the number of molecules per image.

### Quantitative RT-PCR

RNA was isolated from organoids and myofibroblasts using the GeneJET RNA Purification Kit (Thermo Scientific, K0732) according to the manufacturer’s protocol. For qPCR from mouse tissue, RNA was extracted from snap-frozen stomach tissue using the RNeasy Mini Kit (Qiagen, 74104). qPCR was performed with the Power SYBR Green RNA-to-CT 1-Step Kit (Applied Biosystems, 4389986). Reactions were performed in 25 µl containing 50 ng RNA, 12.5 µl SYBR Green mix, 0.2 µl RT mix, and 0.2 µM per primer and run according to the manufacturer’s standard program with additional melt curve step. Relative quantification of gene expression was analyzed with the Step One Software v2.1 (Applied Biosystem) with *Gapdh* as an endogenous reference.

The following mouse primers (Sigma) were used: *Axin2* forward 5′-TGACTCTCCTTCCAGATCCCA-3′, reverse 5′-TGCCCACACTAGGCTGACA-3′; *Bmp1* forward 5′-TGGCCATATCCAGTCTCCCA-3′, reverse 5′-TGTCGTGACGCTCAATCTCA-3′; *Bmp2* forward 5′-GACTGCGGTCTCCTAAAGGTCG-3′, reverse 5′-CTGGGGAAGCAGCAACACTA-3′; *Bmp4* forward 5′-CCGGAAGCTAGGTGAGTTCG-3′, reverse 5′-GGAATGGCTCCATTGGTTCCT-3′; *Bmp5* forward 5′-CAGACCCTGGTACACCTGATG-3′, reverse 5′-CACAGGCACTTCCAGCTAGT-3′; *Bmp6* forward 5′-TGGGATGGCAGGACTGGAT-3′, reverse 5′-TGGCATTCATGTGTGCGTTG-3′; *Bmp7* forward 5′-GGCCTGCAAGAAACATGAGC-3′, reverse 5′-AGTGAACCAGTGTCTGGACG-3′; *Chga* forward 5′-CAAGCACAGAGACGCAGCAG-3′, reverse 5′-GGCTGGTTGGTGATTGGGTA-3′; *Chrdl1* forward 5′-CACTGCCCCAATCGATACCC-3′, reverse 5′-GGTTCTTCTGGGCACACCTTG-3′; *Grem1* forward 5′-AAGTGACAGAATGAATCGCACC-3′, reverse 5′-CCTCAGCTGTTGGCAGTAGG-3′; *Grem2* forward 5′- CATCTCGTCATTGCAGGATGTT-3′, reverse 5′-CGGTTCTTCCGTGTTTCAGC-3′; *Gapdh* forward 5′-TCACCATCTTCCAGGAGCG-3′, reverse 5′-AAGCAGTTGGTGGTGCAGG-3′; *Gast* forward 5′-AGGGGACACCAAGGTGATGA-3′, reverse 5′-AGCAGATTCTGGTGTCGCAG-3′; *Gif* forward 5′-AAGCACAGCGCAAAAACTCC-3′, reverse 5′-GCAACCCCTTCATCCAAAGG-3′; *Id1* forward 5′-GCTCTACGACATGAACGGCT-3′, reverse 5′-AACACATGCCGCCTCGG-3′; *Irf1* forward 5′-ATCTCGGGCATCTTTCGCTT-3′; reverse 5′-TCTGCATCTCTAGCCAGGGT-3′; *Itln1* forward 5′-GTTGCTACCAGAGGTTGCAGT-3′; reverse 5′-AGACCATCTTGTGCCTTTGTGT-3′; *Lgr5* forward 5′-CCTACTCGAAGACTTACCCAGT-3′, reverse 5′-GCATTGGGGTGAATGATAGCA-3′; *Muc5ac* forward 5′-CCTGAGGGTATGGTGCTTGA-3′, reverse 5′-TGTGTTGGTGCAGTCAGTAGAG-3′; *Muc6* forward 5′-CAGCTCAACAAGGTGTGTGC-3′, reverse 5′-GGTCTCCTCGTAGTTGCAGG-3′; *Rspo3* forward 5′-TTGACAGTTGCCCAGAAGGG, reverse 5′-CTGGCCTCACAGTGTACAATACT-3′; *Reg3b* forward: 5′-TCCCAGGCTTATGGCTCCTA-3′, reverse 5′-GCAGGCCAGTTCTGCATCA-3′; *Reg3g* forward: 5′-TCTGCAAGACAGACAAGATGCT-3′, reverse 5′-GCAACTTCACCTTGCACCTG-3′; *Smad6* forward: 5′-CCCTATTCTCGGCTGTCTCC-3′, reverse 5′-CTGGCATCTGAGAATTCACCC-3′; *Oasis* forward: 5′-TCACTGCCCCTCACAAACTG-3′, reverse 5′-TACTCCTTCTTCTTGCGGCG-3′; *Id2* forward: 5′-GCATCCCACTATCGTCAGCC-3′, reverse 5′-AAGGGAATTCAGATGCCTGCAA-3′; *Prx2* forward: 5′-CGTGGCACCAAACGAAAGAA-3′, reverse 5′-TGGAACCAGACTTGGACACG-3′.

qPCR for *Ifn-γ* was performed with TaqMan *Ifn-γ* probe Mm01168134_m1 (Thermo Scientific, 4331182) and TaqMan Fast Virus 1-Step Master Mix (Thermo Scientific, 4444432) according to the manufacturer’s protocol. Relative quantification of gene expression was analyzed with TaqMan *Gapdh* probe Mm99999915_g1 as an endogenous reference.

### Statistics

Data are expressed as mean ± SEM. *P* < 0.05 was considered significant. Differences between two groups were determined by Student’s *t*-test (two-tailed). One-way ANOVA followed by respective post hoc analysis (Tukey’s test) was used for statistical comparison of more than two groups. No statistical methods were used to predetermine sample size. Data are displayed for independent biological samples and at least two independent experiments were performed for the displayed datasets. For data visualization and statistical analysis, GraphPad Prism 8 and R 3.4 software were used.

### Reporting summary

Further information on research design is available in the Nature Research Reporting Summary linked to this article.

## Supplementary information

Supplementary Information

Reporting Summary

## Data Availability

The RNAseq and microarray data generated in this study have been deposited in the National Centre for Biotechnology, Gene Expression Information Omnibus (GEO) under accession code GSE136660. The scRNAseq data generated in this study have been deposited in the National Centre for Biotechnology, Sequence Read Archive (SRA) under accession code PRJNA765498. The previously published scRNAseq data^10^ for validation of our findings are available in the GEO under accession code GSE116514. The source data underlying Figs. 1b, 1e, 1h, 2d, 2e, 2h, 3b, 3d, 3e, 3f, 3h, 4b, 4c, 4d, 4e, 4f, 4g, 4h, 4j, 5a, 5b, 5d, 5f, 5g, 5i, 5j, 5l, 6a, 6b, 6c, 7a, 7b, 7c, 7d, 7f, 7g, 7h, 7j, 8a, 8d, 8e, 8f, 8g, 8h, 8i, 8j, 8l, 8m, 8o, and Supplementary Figs. 1b, 3a, 3b, 3c, 3d, 3e, 3f, 4a, 4b, 4c, 4g, 5a, 5c, 5d, 5e, 5g are provided as a Source Data file. A reporting summary for this article is available as a Supplementary Information file. All other relevant data that support the findings of this study are available from the corresponding authors upon reasonable request. Source data are provided with this paper.

## Source data

Source Data

## Code availability

All computational codes can be accessed under https://github.com/MPIIB-Department-TFMeyer/Kapalczynska_BMP2_IFNy.
