# Supplementary material for: BMP feed-forward loop promotes terminal differentiation in gastric glands and is interrupted by H. pylori-driven inflammation
Source: Nat Commun. 2022 Mar 24;13:1577. doi: 10.1038/s41467-022-29176-w (PMC8948225; doi:10.1038/s41467-022-29176-w)
Supplement: Supplementary file 1 — Supplementary Information [file 41467_2022_29176_MOESM1_ESM.pdf]

Supplementary Information

BMP feed-forward loop promotes terminal differentiation in gastric glands and is interrupted by H. pylori-driven inflammation

Supplementary Figure 1

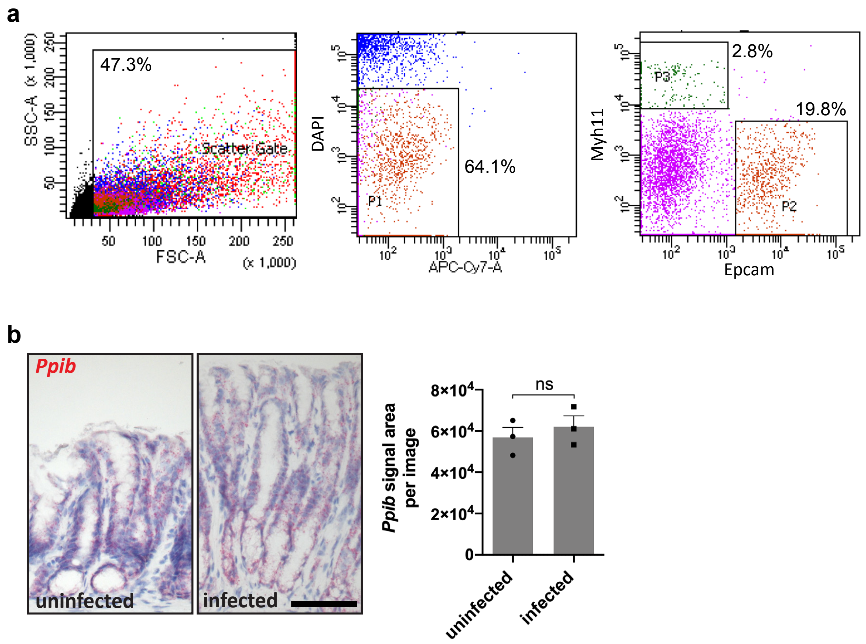

Supplementary Fig.1 Details of FACS sorting for Myh11+ myofibroblasts from uninfected and 2-month *H. pylori* infected *Myh11CreErt2/Rosa26-mTmG* mice.

a) Single viable cells were gated by forward scatter and pulse-width parameters (left), live cells by negative DAPI staining (central) and myofibroblasts by Myh11 signal (right). The gate P3 indicates the cells of interest that were further processed. Note that these cells are Epcam-negative, whereas Epcam+ epithelial cells are gated in P2.
b) Images of *Ppib* (positive control) *in situ* hybridization from antrum tissue of uninfected (n = 3) and 2-month infected mice (n = 3), as well as quantification of the signal.

Images are representative of at least three biological replicates. Scale bar: 100 µm, data are presented as mean ± SEM. Statistical analyses were performed using Student's t-test (two-tailed) for **b**. Source data are provided as a Source Data file.

Supplementary Figure 2

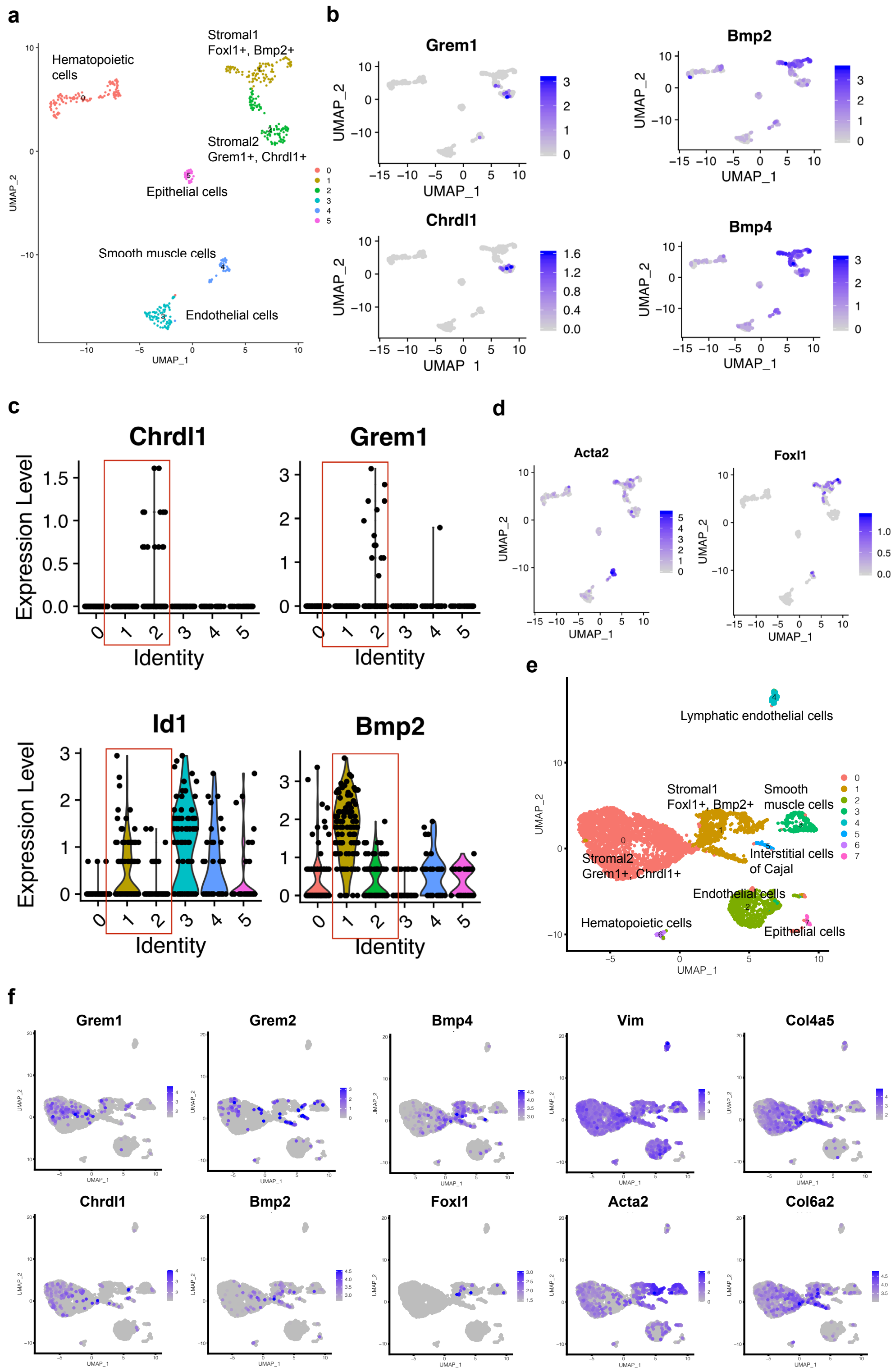

Supplementary Fig.2 Single cell RNAseq from antrum stroma

a) Umap Plot demonstrating specific cell clusters within antrum stromal cells, sorted from the antrum upon dissociation and sorting for Epcam-negative cells.
b) Umap plot depicting expression of selected genes in each cell, note that BMP inhibitors *Grem1* and *Chrdl1* are expressed specifically in the "Stromal 2" population, whereas *Bmp2* is mainly expressed in the "Stromal 1" population.
c) Violin plots for selected genes: *Id1* expression is highly expressed in Stromal 1 population, while Stromal 2 population has much lower *Id1* expression.
d) Feature plot showing expression of selected genes. *Foxl1* was expressed only in Bmp2-expressing cell cluster, while *Acta2* was highly expressed and marked both stromal cell populations.
e) Umap profile from a published scRNA-seq data set from mouse gastric mesenchyme (validation set).
f) Umap plot from the validation data set displaying differential expression of BMP ligands and inhibitors, and mesenchymal markers.

Supplementary Figure 3

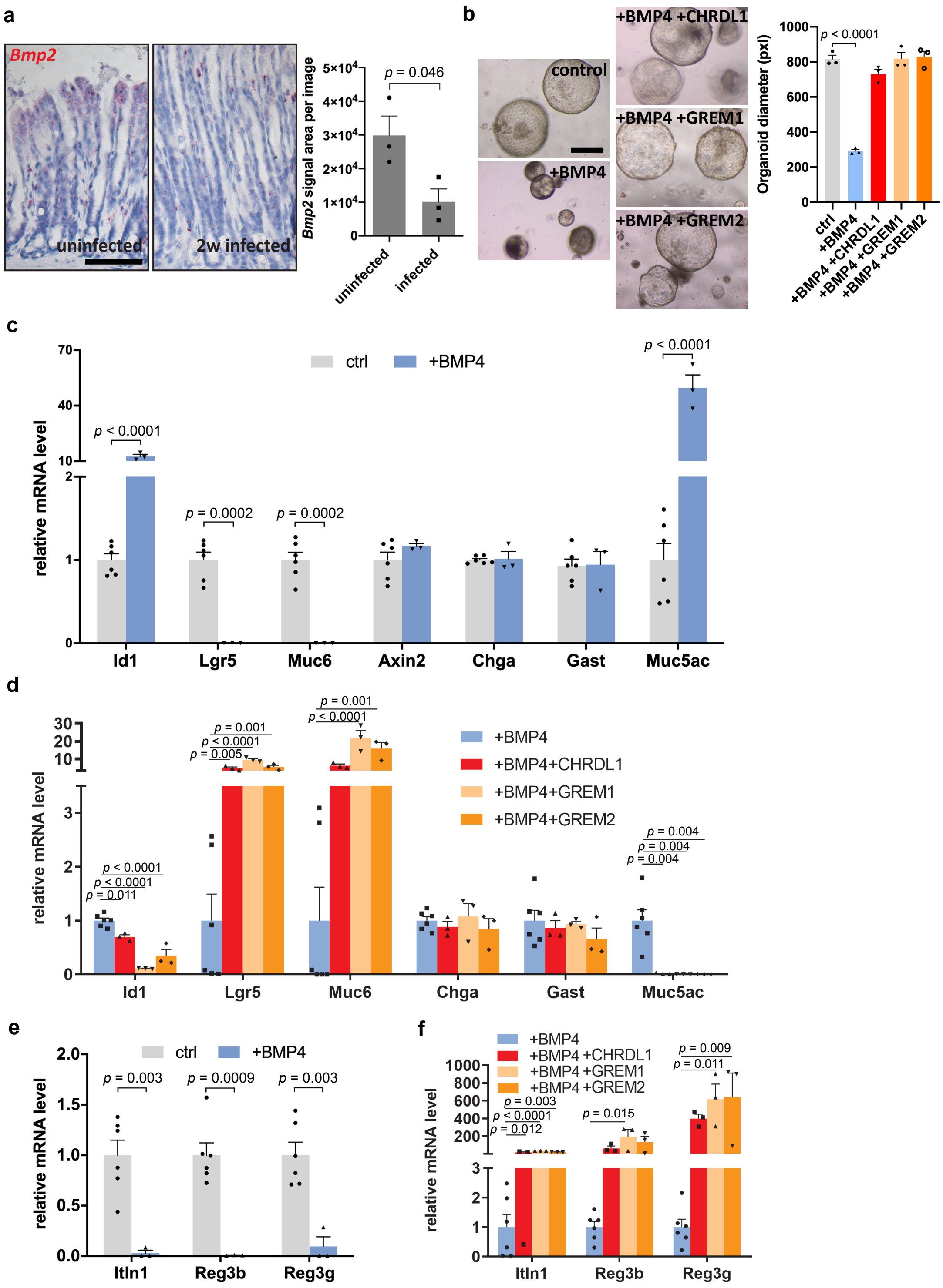

Supplementary Fig.3 The effect of 2-week *H. pylori* effect on *Bmp2* expression and the effect of BMP signaling on cell differentiation

a) Images of *Bmp2* in situ hybridization from antrum tissue from uninfected (n = 3) and 2-week infected mice (n = 3), as well as quantification of the signal. Scale bar: 100 µm.
b) Images of organoids and quantification of the diameter of organoids from antral epithelium either untreated, treated with BMP4 separately, or BMP4 together with BMP inhibitors CHRDL1, GREM1, or GREM2 (n = 3).
c) qPCR for the BMP target gene *Id1* and markers characteristic for specific gland regions from organoids untreated or treated with BMP4 (n = 3).
d) qPCR as in **c** for organoids treated with BMP4 separately or together with the BMP inhibitors CHRDL1, GREM1, or GREM2 (n = 3).
e) qPCR for antimicrobial genes from organoids untreated or treated with BMP4 (n = 3).
f) qPCR for antimicrobial genes from organoids treated with BMP4 separately or together with the BMP inhibitors CHRDL1, GREM1, or GREM2 (n = 3).

Images are representative of at least three biological replicates. Scale bar: 250 µm, except where indicated. Data are presented as mean ± SEM. Statistical analyses were performed using Student's t-test (two-tailed) for **a**, **c**, and **e;** using one-way ANOVA, followed by Tukey's multiple comparisons test for **b**, **d**, and **f**. Source data are provided as a Source Data file.

Supplementary Figure 4

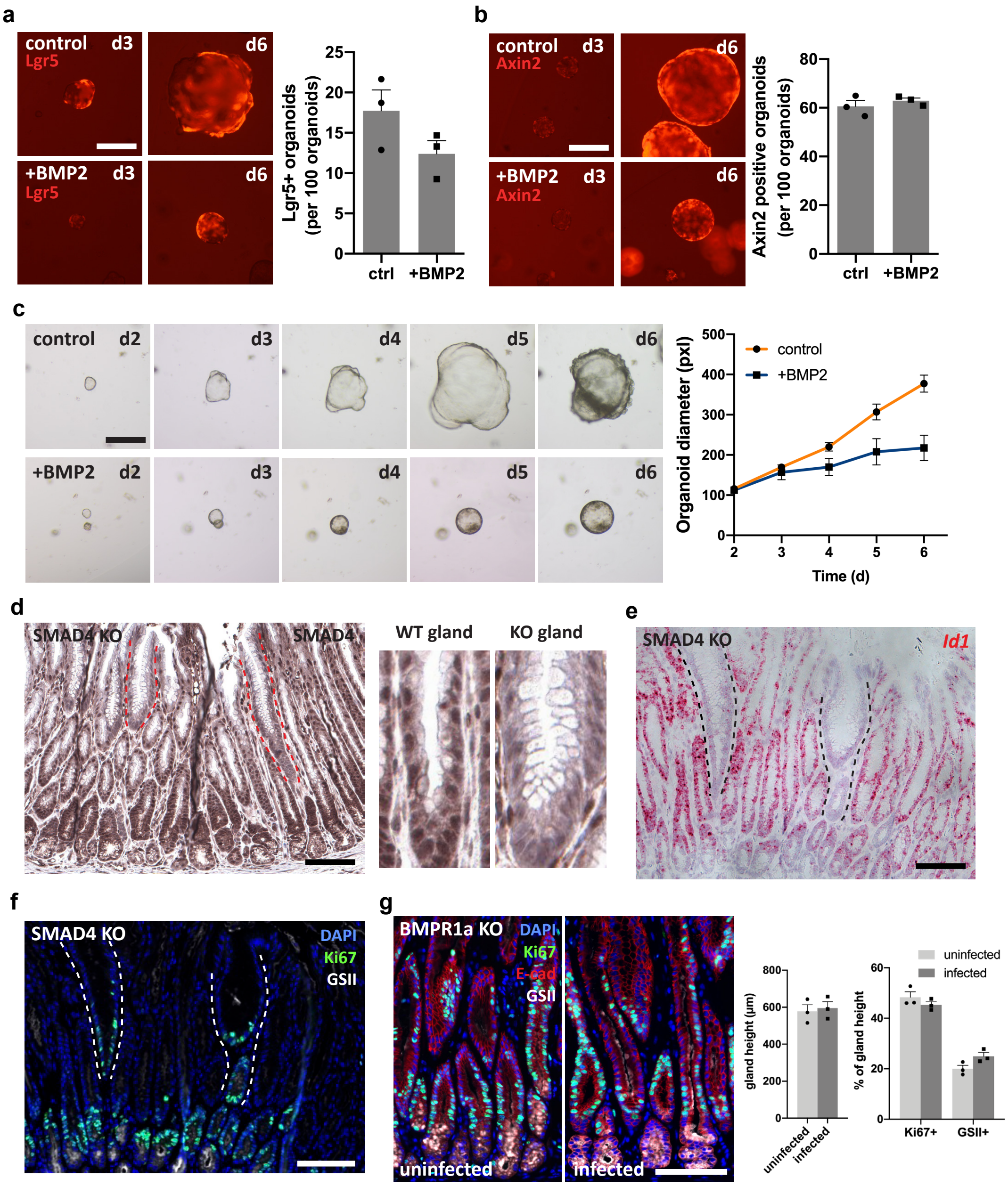

Supplementary Fig.4 BMP signaling induces differentiation of stem cells

a) Lineage tracing for Lgr5 was induced in *Lgr5CreErt2/Rosa26-tdTomato* mice (n = 3). At the same time organoids were treated with BMP2 or left in medium without BMP2. The same organoids were assessed and images from day 3 and day 6 upon treatment are shown. The proportion of tdTomato+ organoids at day 6 was quantified.
b) Lineage tracing for Axin2 was induced in *Axin2CreErt2/Rosa26-tdTomato* mice (n = 3). At the same time organoids were treated with BMP2 or left in medium without BMP2. The same organoids were assessed and images from day 3 and day 6 upon treatment are shown. The proportion of tdTomato+ organoids at day 6 was quantified.
c) Organoids were treated with BMP2 and growth and morphology of the same organoids were assessed daily in comparison to controls (n = 3).
d) Representative image of SMAD4 immunohistochemistry from antrum tissue from *Axin2CreErt2/Smad4*$^{fl/fl}$ mice. Scale bar: 100 µm.
e) Representative image of *Id1* in situ hybridization from antrum tissue from *Axin2CreErt2/Smad4*$^{fl/fl}$ mice. Scale bar: 100 µm.
f) Representative immunofluorescence image of antrum tissue from *Axin2CreErt2/Smad4*$^{fl/fl}$ mice. Scale bar: 100 µm.
g) Immunofluorescence images of antrum tissue from uninfected (n = 3) and 2-month *H. pylori*-infected (n = 3) *Axin2CreErt2/Bmpr1a*$^{fl/fl}$ mice. Gland height of antrum tissue and relative abundance of the Ki67+ and GSII+ compartments were quantified. Scale bar: 100 µm.

Images are representative of at least three biological replicates. Scale bar: 250 µm, except where indicated. data are presented as mean ± SEM. Statistical analyses were performed using Student's t-test (two-tailed) for **a**, **b**, and **g**. Source data are provided as a Source Data file.

Supplementary Figure 5

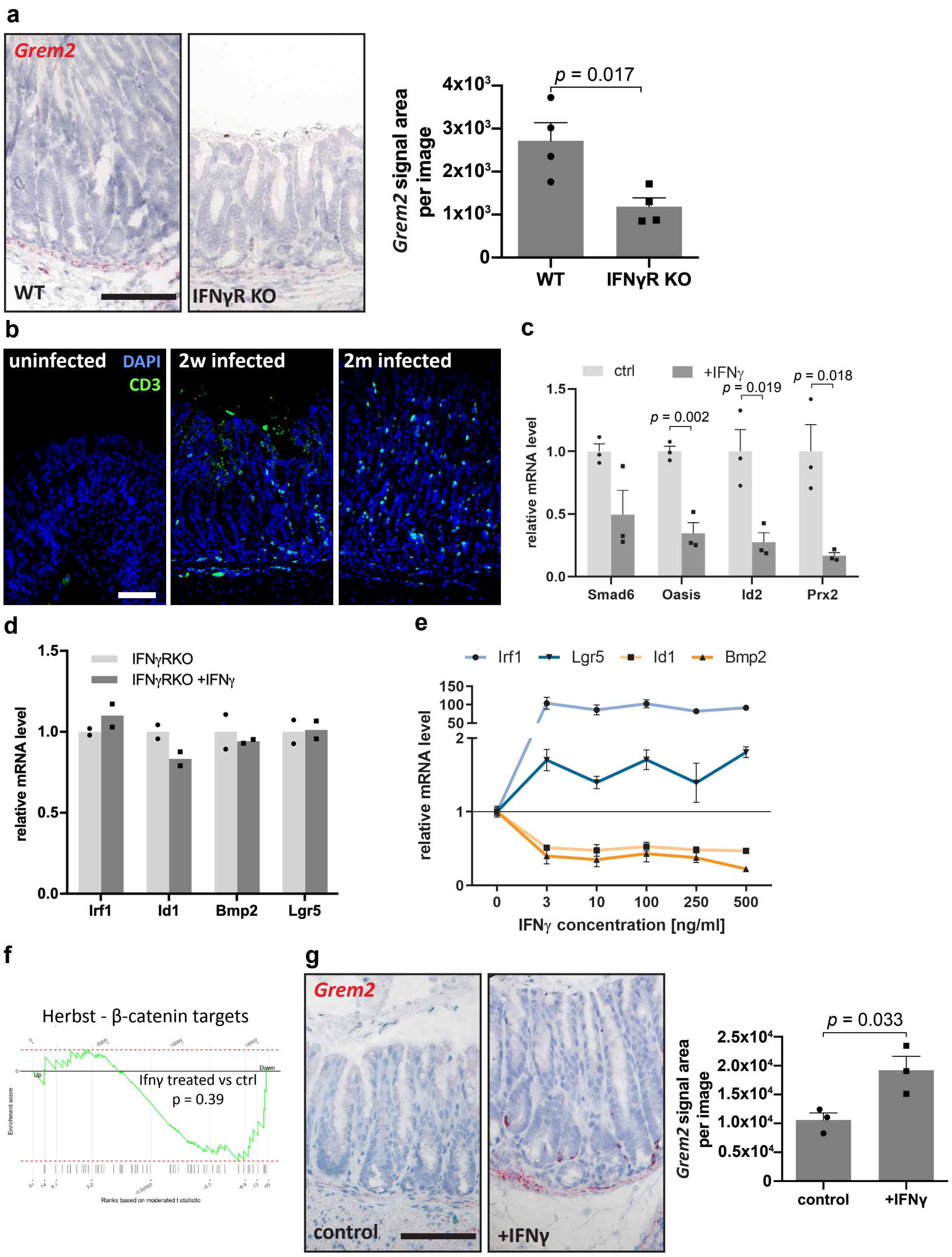

Supplementary Fig.5 Effects of IFN-γ on BMP signaling

a) Single-molecule in situ hybridization for *Grem2* and quantification of *Grem2* signal area per image in antrum tissue from 6-week *H. pylori* infected C57BL/6 mice (n = 4) and *IfnγR* KO mice (n = 4).
b) Immunofluorescence images labeled for CD3 in antrum tissue from uninfected C57BL/6 mice and mice infected with *H. pylori* for 2 weeks and 2 months.
c) qPCR from organoids treated with IFN-γ and untreated controls for selected Bmp target genes (n = 3).
d) qPCR from organoids from *IfnγR* KO mice treated with IFN-γ (n = 2) and untreated controls (n = 2) for several genes that are regulated in wild-type mice.
e) Graph depicting the effects on gene expression induced by IFN-γ using different IFN-γ concentrations (n = 3).
f) GSEA with hallmark of β-catenin target genes for IFN-γ treated organoids compared to untreated ones (n = 2).
g) Single-molecule *in situ* hybridization for *Grem2* and quantification of *Grem2* signal area per image in antrum tissue from untreated mice (n = 3) and mice treated with recombinant IFN-γ for 14 days (n = 3).

Images are representative of at least three biological replicates. Scale bar: 100 µm, data are presented as mean ± SEM. Statistical analyses were performed using Student's t-test (two-tailed) for **a**, **c**, and **g**. Source data are provided as a Source Data file.
